# Supplementary material for: Towards large-scale single-shot millimeter-wave imaging for low-cost security inspection
Source: Nat Commun. 2024 Jul 31;15:6459. doi: 10.1038/s41467-024-50288-y (PMC11291932; doi:10.1038/s41467-024-50288-y)
Supplement: Supplementary file 1 — Supplementary Information [file 41467_2024_50288_MOESM1_ESM.pdf]

Towards Large-scale Single-shot Millimeter-wave Imaging for Low-cost Security Inspection: Supplementary Document

Liheng Bian[1,2*†], Daoyu Li[1†], Shuoguang Wang[3,4†], Chunyang Teng[3], Jinxuan Wu[1], Huteng Liu[3], Hanwen Xu[1], Xuyang Chang[1], Guoqiang Zhao[3], Shiyong Li[3,5*] and Jun Zhang[1*]

[1]MIIT Key Laboratory of Complex-field Intelligent Sensing, Beijing Institute of Technology, 100081, Beijing, China.
[2]Guangdong Province Key Laboratory of Intelligent Detection in Complex Environment of Aerospace, Land and Sea, Beijing Institute of Technology, Zhuhai, 519088, China.
[3]Beijing Key Laboratory of Millimeter Wave and Terahertz Technology, Beijing Institute of Technology, 100081, Beijing, China.
[4]Academy for Network & Communications of CETC, Key Laboratory of Hebei Province on Unmanned System Intelligent Telemetry & Telecontrol Information Technology , 050050, Shijiazhuang, China.
[5]Tangshan Research Institute of Beijing Institute of Technology, 063007, Tangshan, China.

*Corresponding author(s). E-mail(s): bian@bit.edu.cn; lisy_98@bit.edu.cn; zhjun@bit.edu.cn;
†These authors contributed equally to this work.

Contents

1 A review of MMW sparse array synthesis and imaging **1**
- 1.1 MMW near-field imaging based on full arrays 1
- 1.2 Sparse array synthesis for near-field imaging 2
- 1.3 Compressive Sensing (CS)-based reconstruction algorithms for sparse arrays 3
- 1.4 Deep learning-based reconstruction algorithms 4

2 More details about the reported MMW security check framework **6**

3 More details about the statistically sparse array design **7**

4 More details about the physical traits of the statistical importance map **10**

5 More details about the reconstruction and detection methods **14**
- 5.1 The deep learning reconstruction network 14
- 5.2 The untrained learning reconstruction 14
- 5.3 The influence of interpolation in the scattering process 16
- 5.4 Complexity of CCN 17
- 5.5 The detection network 21

6 More details about the transmit and receive antenna isolation and the calibration procedure **25**

7 More comparisons on the test dataset **26**
- 7.1 Additional reconstruction results on the test dataset 26
- 7.2 Additional comparisons among different sparse arrays 28

8 More discussions about applicability and generalization **33**
- 8.1 Clothing material and thickness 33
- 8.2 Body shape 36
- 8.3 Subject position 37
- 8.4 Target position and status 42
- 8.5 Assembly position error of array elements 43

1 A review of MMW sparse array synthesis and imaging

This section provides a brief literature review of Millimeter-Wave (MMW) sparse array synthesis and imaging techniques to further highlight the innovation of the reported technique. The review involves the following several key aspects.

- MMW near-field imaging based on full arrays
- Sparse array synthesis for near-field imaging
- Compressive sensing-based reconstruction algorithms for sparse arrays
- Deep learning-based reconstruction algorithms

1.1 MMW near-field imaging based on full arrays

Here we present a review of recent MMW near-field imaging methods, with a specific focus on full arrays.

We begin with the concept of monostatic arrays. The utilization of a full Single-Input Single-Output (SISO) array entails a substantial number of elements, thereby resulting in prohibitively high manufacturing costs. Consequently, mechanically scanning linear array systems have found widespread application in conjunction with the Range Migration Algorithm (RMA) [S1,S2] to facilitate large-scale imaging. Key works in this domain include ref. [S3–S8].

However, two main challenges are hindering the mechanically scanning linear array system's wide adoption. First, it is hard to enable high-throughput imaging due to the time-consuming scanning procedure. In addition, small movements of subjects during scanning will affect the imaging and detection accuracy. In practice, it is challenging to ensure complete steadiness, especially for elderly individuals. Slight movements, such as body swaying or shaking, can significantly degrade image quality. The blurred images caused by even slight movements of subjects [S9] can compromise the accuracy of subsequent concealed object detection algorithms [S10]. As shown in Fig. S1, when the subject experiences a jiggle during scanning, it becomes almost impossible to achieve accurate imaging and detection. Similarly, if there is displacement of body parts during the scanning process, the imaging and detection of the displaced area will be affected. On the other hand, the electronic scanning framework offers short imaging time, and even with motion, it has minimal impact on imaging and target recognition.

In contrast, MIMO arrays offer the advantage of data collection through a single snapshot, necessitating fewer elements [S11]. A typical MIMO imaging prototype involves square clutters [S12], wherein the computationally intensive back-projection algorithm [S13] is employed for image reconstruction. Several Fourier-based imaging algorithms have been developed for acceleration [S11, S14–S17]. However, these methods struggle to achieve real-time imaging due to the higher-dimensional processing when compared to SISO arrays. Phase calibration methods can be employed to attain real-time imaging but may introduce image distortions [S18, S19]. Consequently, the cost-efficient MIMO

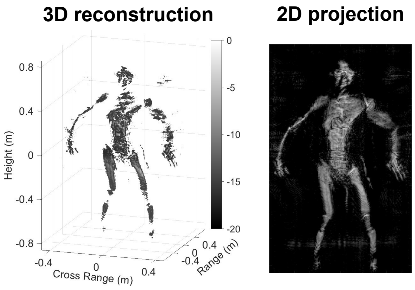

Fig. S1: The imaging results when the subject experiences a jiggle.

arrays often confront challenges in simultaneously achieving fast and high-quality imaging.

A parallel avenue of exploration involves the amalgamation of linear or arc MIMO arrays with mechanical scanning structures (MIMO-SAR) for enhanced cost-effectiveness. MIMO linear arrays can be configured to scan along straight [S20–S22] or circular tracks [S23–S25]. In a notable departure from this, the transformation from arc arrays to equivalent linear arrays has been proposed [S26] to enable azimuthal imaging for circular-arc MIMO arrays. Furthermore, a full wavenumber domain 3D imaging algorithm [S27] has been devised within this framework. However, akin to MIMO arrays, MIMO-SAR schemes grapple with protracted imaging times attributed to the higher-dimensional processing. Moreover, they are unable to achieve one-shot imaging due to mechanical scanning.

1.2 Sparse array synthesis for near-field imaging

Imaging with full arrays demands large-scale arrays, resulting in high costs. Sparse Array Synthesis (SAS) offers a solution to this challenge. We delve into existing SAS literature to provide a brief overview, with relevant references in Tab. S1.

Table S1: Recent advancements in sparse array synthesis methods for MMW near-field imaging

Ref	Scale(m)	Elements	Array Form	Distance(cm)	Band(GHz)	Step(MHz)	Sparsity(%)	PSL(dB)	Year
[S28]	0.5	20	linear	100	10.0-18.0	-	-	-	2008
[S29]	0.425×0.425	20	planar	75	5-25	-	-	-	2009
[S20]	0.5	12	linear (SAR)	50	2.8-19.5	-	23.5	-	2010
[S30]	0.4×0.4	25	planar	50	3-19.5	-	-	-18	2010
[S23]	0.65	58	linear (SAR)	60	75-90	117	20	-	2011
[S31]	0.5×0.5	25	planar	50	9.5-12.5	-	-	-22	2012
[S32]	0.707	26	curvilinear (SAR)	31	56-62	400	-	-	2013
[S33]	0.48×0.48	20	planar	50	2.14-9.04	-	-	-19.04	2015
[S34]	0.5×0.5	20	planar	50	2.55-7.89	-	-	-	2016
[S35]	0.4×0.4	6	planar	28	77.38-80.93	-	-	-	2019
[S36]	0.425×0.425	24	spiral	60	3.5-8.5	-	-	-17.7	2020
[S37]	3	6	linear	400	1-3	17.9	-	-	2021
[S38]	0.5×0.5	68	T-shaped	120	90-96	68	33.7	-	2023
[S39]	0.6×0.6	3732	planar	50	30-35	50	25.5	-	2023
Our	**2.0×1.0**	**7998**	planar	40	32-37	100	10.0	-	2024

Several works [S29, S31, S40] have proposed sparse arrays with fixed array topologies. However, it is important to acknowledge that these topological configurations are bound by practical constraints and exhibit limited flexibility. Some works have employed optimization techniques, such as the Simulated Annealing algorithm (SA) [S32] and the Particle Swarm Optimization (PSO) method [S28], to achieve SAS for near-field imaging.

To address the challenges posed by the stochastic optimization methods, researchers have focused on designing sparse array topologies that capitalize on principles of uniformity and reduced element shadowing [S33, S34]. Although these designs have yielded arrays with favorable focusing and sidelobe-suppression properties, stochastic optimization approaches are computationally intensive and often suffer from local convergence particularly when executed over limited random trials. On the contrary, convex optimization methods [S38, S39] offer a promising avenue for SAS. However, it's hard to apply the optimization-based design methods for very large arrays (such as the reported 430×186-element array), given the substantial computational burden they impose in terms of both time and memory requirements. Moreover, while the previously mentioned methods demonstrate satisfactory performance with relatively limited target scales, they may not yield favorable reconstructed images for larger targets.

In contrast to the existing paradigms of manually crafted and optimized arrays, our research reports a novel statistically sparse array design method. This approach starts by collecting a set of real-captured echoes through a full-sampled antenna array and subsequently analyzing the statistical importance ranking map of array elements. Theoretically, a higher value within this ranking map indicates the significance of an element within the antenna array. Guided by this ranking map, we experimentally derive a statistically optimized sampling strategy to reduce the number of inefficient elements. This innovative statistical methodology does not rely on handcraft design or resource-consuming optimization which are hard to employ on large-scale array design, achieving high-quality imaging for a large-scale (430×186-element) array.

1.3 Compressive Sensing (CS)-based reconstruction algorithms for sparse arrays

Sparse array systems employ specific algorithms to achieve effective image reconstruction. Several works have explored the application of various CS-based techniques to acquire 2D images [S41–S46]. Although CS algorithms have been applied in sparse Inverse Synthetic Aperture Radar (ISAR) imaging [S45, S46] and two-dimensional scenarios [S42], it is essential to acknowledge that persistent challenges related to high memory consumption and computational complexity have not yet been fully addressed. Furthermore, these methodologies typically prove inadequate when it comes to reconstructing meaningful 3D images. Efforts to develop 3D image reconstruction techniques based on CS principles are presented in [S47–S49].

Specifically, one avenue of exploration involves the utilization of the Diffraction Tomography (DT) method [S47] and the Two-Level Block Matching Pursuit (TLBMP) algorithm [S48] in conjunction with CS for 3D Through-the-Wall Radar Imaging (TWRI). Unfortunately, these approaches have been associated with challenges such as low resolution and protracted reconstruction times. Furthermore, an interpolation-free holographic imaging algorithm has been developed as part of the CS iterative optimization process [S50]. Nevertheless, comparative evaluations have revealed that this algorithm fails to achieve comparable reconstruction results under similar conditions.

In contrast to these methodologies, our study introduces the physics-informed untrained learning approach, which enables accurate and robust MMW reconstruction from sparsely sampled echoes. Instead of training-based techniques, it optimizes a lightweight complex-valued network using an objective function rooted in the physical model of MMW scattering. Experiments showcase the superior performance of our approach, manifesting an average 2.61dB and 4.19dB improvement in PSNR compared to existing CS-based approaches at 10% and 25% sampling ratios, respectively. Moreover, our technique exhibits pronounced advantages in the context of detecting concealed targets from sparsely sampled echoes.

1.4 Deep learning-based reconstruction algorithms

With the bloom of Deep Learning (DL) theory, various DL-based reconstruction methods (listed in Tab. S2) have been proposed to yield high-quality images within specific datasets. It is important to note that some metrics may vary greatly in different imaging scenarios and datasets, due to the varying complexity of imaging different scenes. The effectiveness and reliability of these approaches should be interpreted in specific imaging conditions and dataset characteristics.

In this domain, many researchers have employed various neural networks [S51–S53] to enhance the imaging results of pre-reconstruction algorithms. However, these methods do not fully utilize the anisotropic scattering characteristics of targets and the physical process of scattering. Moreover, conventional networks are often considered black boxes. To enhance interpretability and robustness, some works combined model-based CS methods and neural networks for reconstruction [S54, S55, S57].

On the other hand, deep learning techniques have been directly employed for dealing with the Inverse Scattering Problem (ISP) end-to-end, where these methods unfold the nonlinear electromagnetic inverse scattering process using deep neural networks, thereby reducing computational costs [S63–S65, S67, S70, S75, S81]. Besides, CS methods can also be integrated into deep learning networks to achieve ISP [S72, S74, S76, S80]. However, some researchers adopt transfer learning by employing optical data as a reference to enhance the reconstruction results [S81], but this may hinder learning the penetrability characteristics of electromagnetic waves, which are vital for concealed object detection.

Table S2: Recent advancement of learning-based MMW imaging techniques. The light yellow background represents the enhancement methods, where the input is a pre-reconstructed image and the output is the enhanced image. The pink background represents the end-to-end methods, where the input is an EM wave signal and the output is the reconstructed image.

Ref	Train/Test	Network	2D/3D	Scenario	MSE	PSNR	SSIM	FA	TCR	ENT	IC	Year
[S51]	-/-	CVNN	2D	Terrain	377	16.05	-	-	-	-	-	2016
[S52]	50000/-	CV-CNN	2D	Point scatters	-	-	-	-	-	-	-	2018
[S53]	600/-	UNet	2D	Airplane scatters	-	-	-	30	67.37	4.76	12.54	2019
[S54]	-/-	ADMM-CNN	2D	Airplane scatters	-	23.87	-	-	-	-	-	2020
[S55]	5184/576	CS-CNN	2D	Point scatters	-	-	-	-	-	-	-	2020
[S56]	420/60	SARNet	2D	Deer	-	22.98	0.84	-	-	-	-	2021
[S57]	10000/-	GAN	2D	Point scatters	-	26.64	0.84	-	-	-	-	2022
[S58]	1520/80	CVPHD	2D	Yak-42 aircraft	-	-	0.7989	-	-	-	-	2022
[S59]	5000/-	AGAN	2D	Point scatters	-	44.77	-	-	-	8.750	-	2022
[S60]	2082/520	UFGAN	2D	Yak-42 aircraft	-	-	0.8163	-	91.20	-	-	2022
[S61]	1000/100	MF-ADMM-Net	2D	Sea surface	-	-	0.91	-	-	6.33	-	2022
[S62]	8920/1000	PMANN	2D	Characters	-	34.79	0.996	-	-	-	-	2023
[S63]	7000/2000	CNN	2D	Letter-shaped objects	0.0447	-	0.8685	-	-	-	-	2018
[S64]	475/25	UNet-CNN	2D	Circular-cylinders	-	-	-	-	-	-	-	2018
[S65]	475/25	UNet	2D	Cylinders	-	-	-	-	-	-	-	2020
[S66]	-/-	CIST	2D	Yak-42 aircraft	-	-	-	-	22.57	0.2418	-	2020
[S67]	800/200	2D-ADMM-Net	2D	Yak-42 aircraft	-	45.58	0.99	-	-	0.3194	-	2021
[S68]	1000/-	RMIST-Net	3D	Point scatters	-	45.14	-	-	-	-	-	2021
[S69]	-/-	AF-AMPNet	2D	Airplane scatters	0.61	-	0.978	-	-	6.563	63.52	2021
[S70]	2223/-	U-ADMMNet	2D	Satellite model	-	38.22	-	-	-	-	-	2021
[S71]	10000/-	TPSSI-Net	3D	A cone scatter model	-	-	0.84	-	-	-	-	2021
[S72]	36000/4000	MDLI-Net	2D	Point scatters	-	-	0.9998	-	-	-	-	2021
[S73]	1000/-	LFIST-Net	3D	Fighter point cloud model	-	41.71	0.997	-	-	-	-	2021
[S74]	300/70	LRSR-ADMM-Net	2D	Complex surface features	-	23.99	0.8261	-	-	-	-	2022
[S75]	4537/1134	DNN	2D	Car model	-	25.34	0.9607	-	-	-	-	2022
[S76]	12000/-	ATResCS	2D	Wheat	-	43.82	0.957	-	-	-	-	2022
[S77]	-/-	SAF-3DNet	3D	Rabbit point cloud model	-	47.78	0.9924	-	-	-	-	2022
[S78]	2000/-	SISR-Net	3D	Vehicle point cloud model	-	35.74	0.704	-	-	-	-	2022
[S79]	1000/-	SFH-ADMM-Net	3D	Fighter point cloud model	-	38.70	0.972	-	-	-	-	2022
[S80]	600/400	2D-IADIANet	2D	Point scatters	-	34.75	0.969	-	-	-	-	2023
[S81]	-/-	GAN	2D	Airplane scatters	-	28.60	0.86	-	-	-	-	2023

In the realm of 3D imaging based on deep learning, scholars have proposed dedicated learning-based networks [S71, S77, S78] or integrated CS methods into the frameworks [S68, S73]. However, all the aforementioned methods often heavily rely on training datasets, which can result in overfitting issues and a lack of generalization and robustness. Therefore, further research is needed to address these challenges and improve the performance of 3D imaging algorithms in real-world scenarios.

In contrast, our proposed untrained learning scheme eliminates the need for conventional training procedures and avoids the pattern transfer process, preserving the inherent penetrability properties of MMW. The reported technique offers superior reconstruction performance compared to existing CS or DL methods.

In summary, we present a large-scale, single-shot MMW imaging framework leveraging a sparse antenna array. This framework facilitates cost-effective yet high-fidelity security inspections while incorporating an interpretable learning scheme. Furthermore, the scheme delivers accurate and robust image reconstruction from sparsely sampled echoes. Finally, a neural network is developed for automatic object detection, and experimentally demonstrated successful detection of concealed centimeter-sized targets using 10% sparse array, whereas all the other contemporary approaches failed at such a low sampling ratio.

2 More details about the reported MMW security check framework

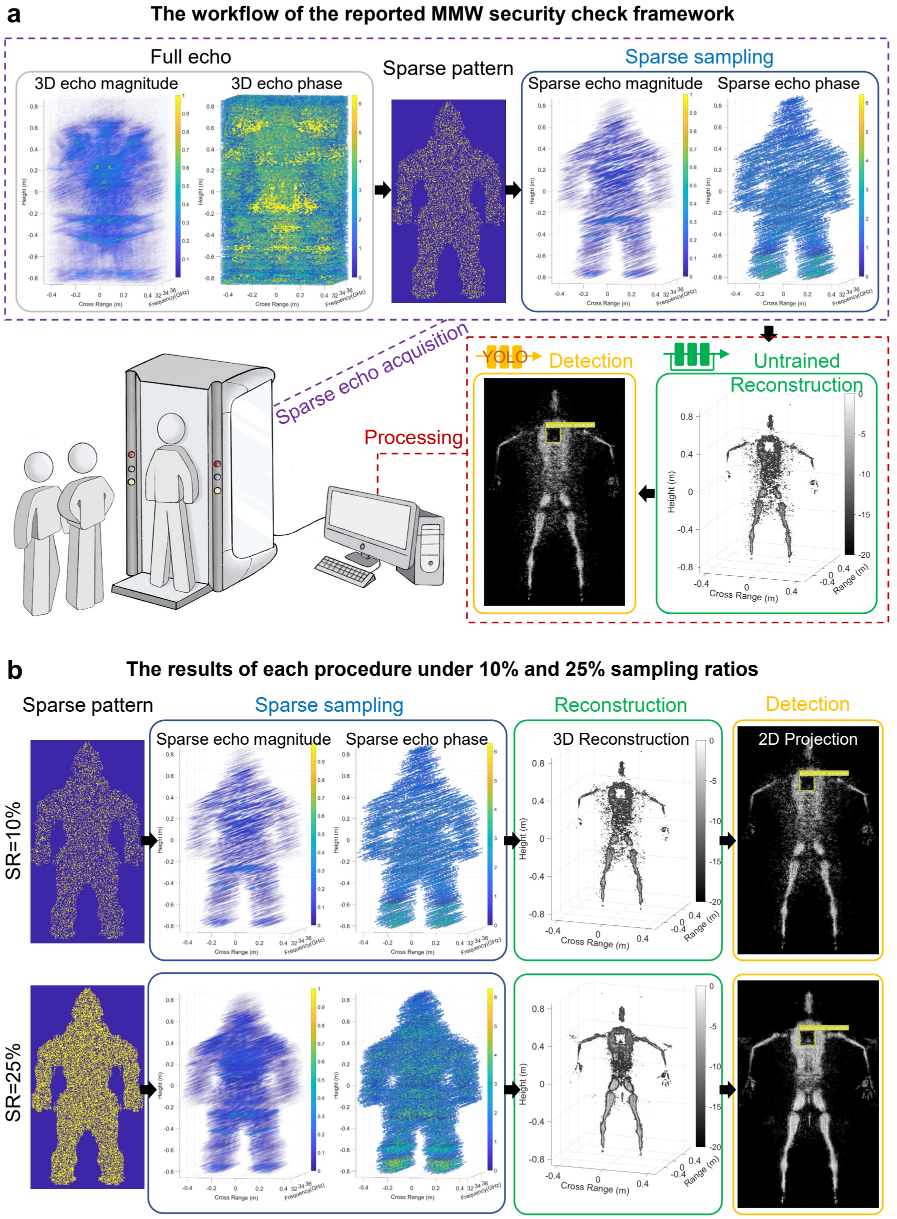

Fig. S2: The workflow of the reported MMW security check framework (**a**) and the results of each procedure under 10% and 25% sampling ratios (**b**).

We summarize the workflow of the reported MMW security check framework as shown in Fig. S2. The framework consists of three main techniques:

1. Sparse echo acquisition: We first acquire the sparse echo of the subject using the statistically optimized sparse antenna array.
2. Untrained reconstruction: We reconstruct the 3D MMW image from the sparse echo using the untrained learning method.
3. Detection: We finally apply the YOLO network to detect the concealed targets with the reconstructed image.

Above all, we have established a complete MMW security check workflow encompassing sparse echo acquisition, reconstruction, and detection. Based on this framework, it is promising to achieve a highly reliable, low-cost, and high-throughput MMW security check system.

3 More details about the statistically sparse array design

We summarize the overall workflow to obtain a statistically sparse array design M in Fig. S3 and as follows:

- **Collecting the echo dataset**: This workflow starts by collecting the 3D echo dataset. We have collected a large-scale 3D echo dataset which contains 1934 human security inspection echoes. Then we extract the amplitude and phase from the center-frequency cross-sections of 3D echoes.
- **Obtaining the statistical importance map**: The statistical importance map $\bar{M}$ is the production of the averaged amplitude ($\bar{A}$) and inverse phase gradient ($\bar{P}$) of 2D cross-sections. This approach was adopted as an intuitive means of statistically integrating the significance of both amplitude and phase components. The averaged representations $\bar{M}$, $\bar{A}$, and $\bar{P}$ shown in Fig. S3, offer a more comprehensive portrayal of the outline and structural characteristics of all the subjects being tested. A higher value in $\bar{M}$ denotes higher statistical importance of the element in the array.
- **Generating the sparse array design**: Next, we perform Monte Carlo sampling to generate the sparse array design M. All element positions are assigned an importance order, where this importance order is the sorting of the element positions on $\bar{M}$ from the highest value to the lowest value. Generate a uniformly random function $r(n)$, where the horizontal axis is the importance order ($n = 1 \to N$, with 1 being most important and N being least important), and the vertical axis takes values from 0 to 1. Given a threshold S, search from 1 to N for n where $r(n) > S$, until the number meets the preset sampling ratio. These positions where $r(n) > S$ correspond to the retained positions in the sparse array design M. When 1-S equals to the sampling ratio, the obtained array is a uniformly random array. There won't be enough elements in the resulting sparse array when 1-S is less than the sampling ratio.

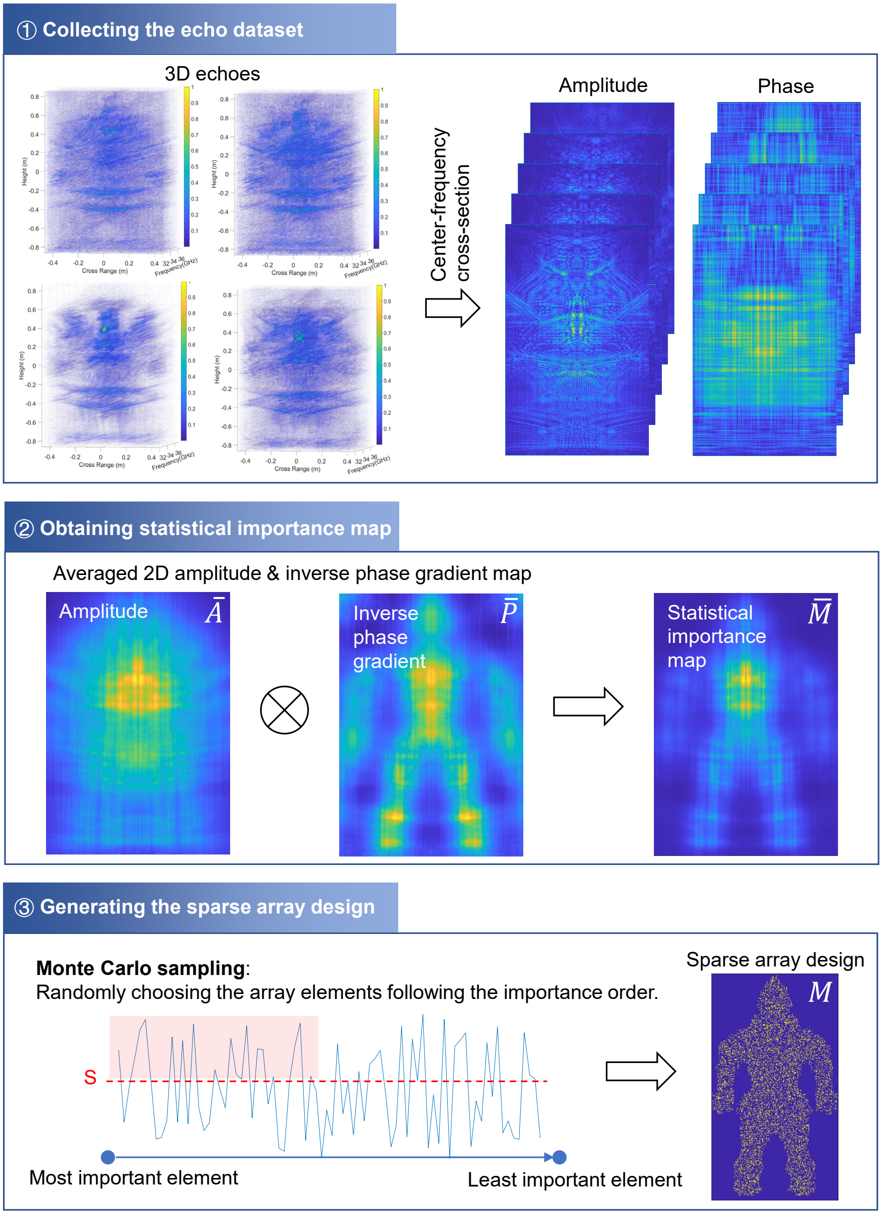

Fig. S3: The workflow for generating a statistically sparse array design. We start by collecting the 3D echo dataset and then extracting the center-frequency cross-sections of these echoes. The statistical importance map $\bar{M}$ is the production of averaged amplitude map $\bar{A}$ and inverse phase gradient map $\bar{P}$. The sparse array design is derived from M by Monte Carlo sampling.

- **Determining the optimal sparse array**: Different hyperparameter S correspond to different M, so we evaluated the performance of different M under 10% and 25% sampling ratios. By numerical comparisons of randomly selected echoes (Fig. S4), the optimized statistical sparse arrays at 10% and 25% sampling ratios correspond to S equals 0.8 and 0.5, respectively.

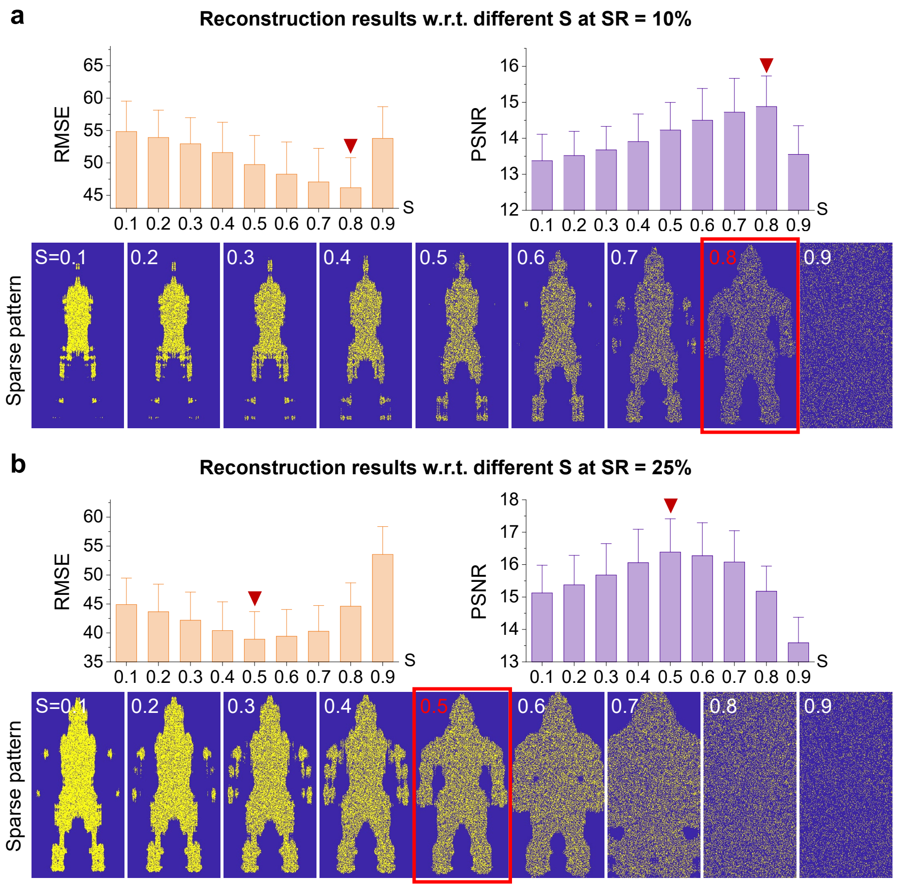

Fig. S4: Numerical comparisons of reconstruction accuracy w.r.t. different S when the sampling ratio equals 10% (**a**) and 25% (**b**). The error bar represents the standard deviation. The optimal sparse patterns of SR=10%&25% correspond to $S = 0.8\&0.5$, respectively.

4 More details about the physical traits of the statistical importance map

Here, we present a theoretical analysis to explain the principle of statistical importance ranking of MMW echoes. The scattering characteristics of targets do not vary significantly within the frequency band of the system, as shown in Fig. S5. Therefore, we opt for the center-frequency cross-sections to reflect the overall echoes. The following analysis involves the physical traits for the reported sparse array design method: higher values in amplitude $\bar{A}$, inverse phase gradient $\bar{P}$, and importance map $\bar{M}$ correspond to higher significance.

Amplitude $\bar{A}$

The amplitude of the echo distribution plays a vital role as an indicator of significance within the array. The MMW imaging system's echo can be mathematically expressed as:

$$s\left(x', z', k\right) = \int\int\int \sigma\left(x, y, z\right) e^{-\mathrm{j}k\sqrt{\left(R_0 - y_0\right)^2 + \left(x' - x\right)^2 + \left(z' - z\right)^2}} \mathrm{d}x\mathrm{d}y\mathrm{d}z. \tag{S1}$$

where $\sigma\left(x, y, z\right)$ represents the scattering coefficient, and k denotes the wavenumber. The variables x' and z' denote the antenna location along the azimuth and height, respectively, and R_0 represents the distance from the target to the array.

Thus, a larger amplitude $\left\| s\left(x', z', k\right) \right\|$ indicates a larger $\sigma\left(x, y, z\right)$ within the beam coverage, corresponding to a higher average reflection. Consequently, a higher value in $\bar{A}$ denotes a higher importance ranking for the corresponding array element.

Inverse phase gradient $\bar{P}$

To simplify the problem, we first discuss the one-dimensional case where the target is distributed along the x axis. Assuming that the Born approximation [S82] is satisfied during the scattering process with omitted propagation attenuation, the echo can be obtained as:

$$s\left(x'\right) = \int \sigma\left(x\right) e^{-\mathrm{j}k_0\sqrt{R_0^2 + \left(x' - x\right)^2}} \mathrm{d}x, \tag{S2}$$

where k_0 denotes the wavenumber of the working single-frequency signal. Considering narrow antenna beampattern, we have $\left\| x' - x \right\| \ll R_0$, then keep the first two terms of the Taylor expansions of the expressions under root, Eq. (S2) can be rewritten as:

$$
\begin{aligned}
s\left(x'\right) &\approx \int \sigma\left(x\right) e^{-\mathrm{j}k_0\left[R_0+\frac{\left(x'-x\right)^2}{2R_0}\right]} \mathrm{d}x \\
&= e^{-\mathrm{j}k_0 R_0} \int \sigma\left(x\right) e^{-\mathrm{j}k_0\frac{\left(x'-x\right)^2}{2R_0}} \mathrm{d}x.
\end{aligned}
\tag{S3}
$$

The gradient along the x-axis can be expressed as:

$$
\frac{\mathrm{d}s\left(x'\right)}{\mathrm{d}x'} = C\int \sigma\left(x\right) e^{-\mathrm{j}k_0\frac{\left(x'-x\right)^2}{2R_0}} \left(2x'-2x\right) \mathrm{d}x,
\tag{S4}
$$

where

$$
C = \mathrm{j}\frac{k_0}{2R_0} e^{-\mathrm{j}k_0 R_0}.
\tag{S5}
$$

The element can be illuminated when its beamwidth covers the target. The gradient item can be rewritten as:

$$
\frac{\mathrm{d}s\left(x'\right)}{\mathrm{d}x'} = -2C\int_{x'-x_{\max}}^{x'+x_{\max}} \sigma\left(x\right) e^{-\mathrm{j}k_0\frac{\left(x'-x\right)^2}{2R_0}} \left(x-x'\right) \mathrm{d}x,
\tag{S6}
$$

where

$$
x_{\max} = R_0 \tan\frac{\Theta_h}{2},
\tag{S7}
$$

Θ_h represents the antenna beamwidth. By employing the integration by parts method, we can derive the following expression:

$$
\begin{aligned}
\frac{\mathrm{d}s\left(x'\right)}{\mathrm{d}x'} = &\frac{2R_0 C}{k_0}\left\{ e^{-\mathrm{j}k_0\frac{x_{\max}^2}{2R_0}} \left[\sigma\left(x'-x_{\max}\right) - \sigma\left(x'+x_{\max}\right)\right] + \right. \\
&\left. \int_{x'-x_{\max}}^{x'+x_{\max}} \sigma'\left(x\right) e^{-\mathrm{j}k_0\frac{\left(x'-x\right)^2}{2R_0}} \mathrm{d}x \right\}.
\end{aligned}
\tag{S8}
$$

The first row in Eq. (S8) illustrates the influence of the target amplitude near the boundary of the antenna beamwidth. The second row in the same equation illustrates the impact of target fluctuations. Due to the relatively uniform distribution of scattering coefficients $\sigma(x, y)$ of human targets, both of these terms tend to approach zero, specifically $\frac{\mathrm{d}s\left(x'\right)}{\mathrm{d}x'} = 0$. Conversely, in situations where the target fluctuates or there's a void in the imaging area dominated by echo noise, both terms in Eq. (S8) deviate from zero. Under these conditions, the accumulation of terms in the second row of Eq. (S8) can result in a significant $\frac{\mathrm{d}s\left(x'\right)}{\mathrm{d}x'}$.

The analysis presented above leads to the conclusion that selecting elements with small gradients can enhance the quality of the illumination for the specific target of interest. That is, a larger value in $\bar{P}$ indicates that the corresponding element has a higher importance ranking.

Importance map $\bar{\boldsymbol{M}} = \bar{\boldsymbol{A}} \times \bar{\boldsymbol{P}}$

The statistical importance map $\bar{M} = \bar{A} \times \bar{P}$ reflects both the amplitude and phase distribution of the echo dataset. Similarly, a larger value in $\bar{M}$ indicates a higher importance of the corresponding element in the array. Following the workflow in Fig. S3, the sparse array design is generated. We can adjust the hyperparameter S to find a suitable coverage range and sparsity of the sampling pattern.

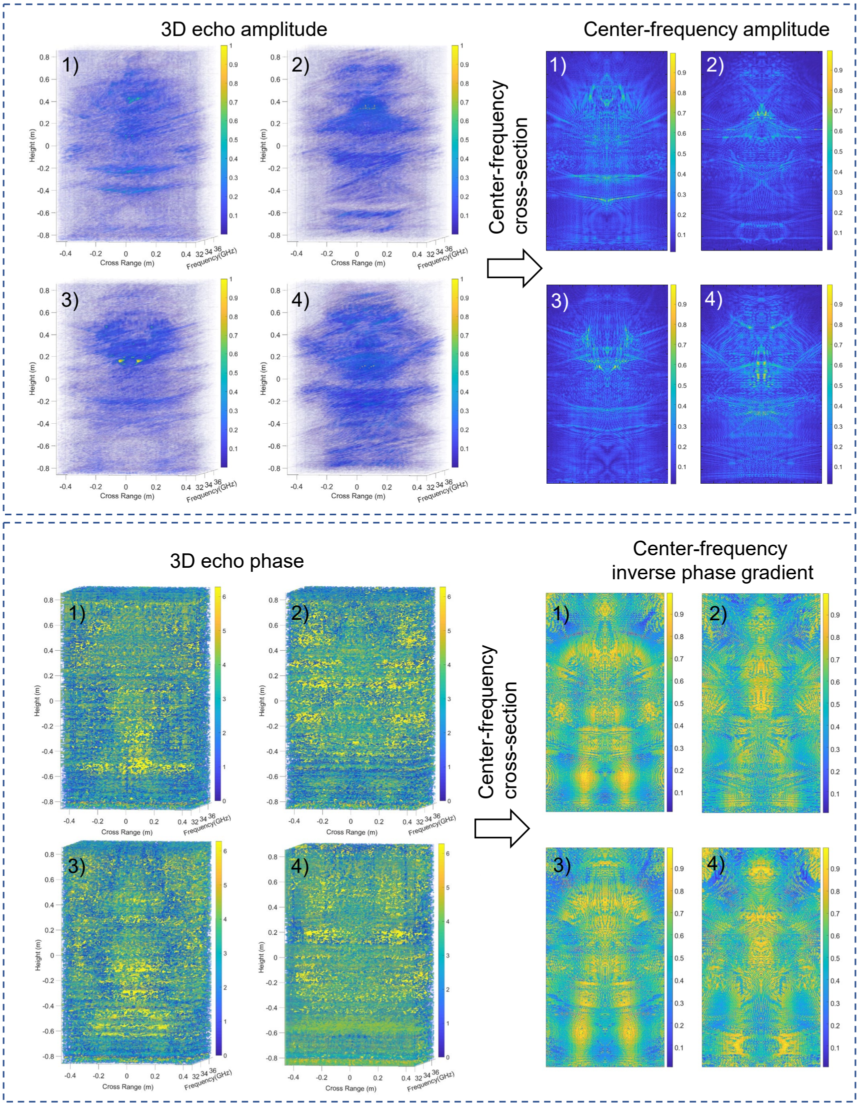

Fig. S5: The exemplar 3D echo amplitudes and phases, and the corresponding center-frequency amplitude and inverse phase gradient cross-sections.

5 More details about the reconstruction and detection methods

5.1 The deep learning reconstruction network

Figure S6 shows the architecture of the learning-based reconstruction network mentioned in the main text. It is a complex-valued UNet-like network, including an encoder, a decoder, and an output layer. Considering the scale of the training set ($\sim 1 \times 10^3$ samples) and the complexity of large-scale security imaging, we applied the enhancing networks that take the 3D initialization reconstructed by RMA as input and produce enhanced 3D reconstructions. The encoder consists of 5 layers of Complex Conv (kernel size=3, stride=2, padding=1) + Complex BN + ReLU. The output channels of these layers are 64, 128, 256, 512, and 1024, respectively. The decoder consists of 5 layers of Complex TransConv (kernel size=4, stride=2, padding=1) + Complex BN + ReLU. The output channels of these layers are 1024, 512, 256, 128, and 64, respectively. The output layer is a complex-valued Conv layer (kernel size=1, stride=1, padding=0). We trained four networks on reconstructions of 10% random array, 10% statistically sparse array, 25% random array, and 25% statistically sparse array, respectively. We applied the AdamW [S83] solver with a 0.001 learning rate and the cosine annealing schedule [S84]. Each network was trained for more than 1000 epochs to ensure convergence.

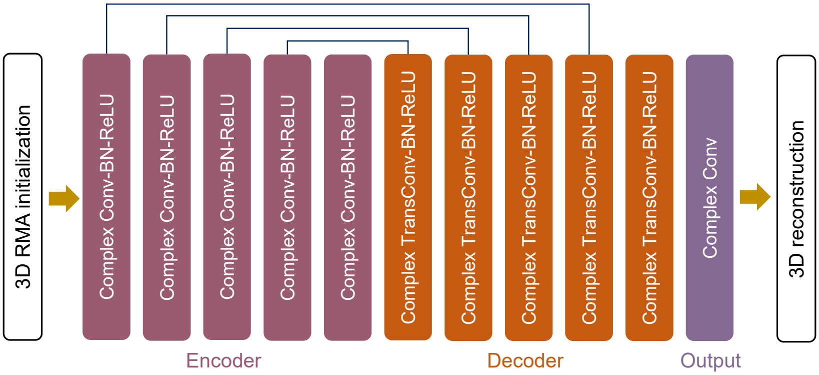

Fig. S6: Architecture of the learning-based reconstruction network.

5.2 The untrained learning reconstruction

We introduce the untrained learning-based reconstruction into 3D MMW imaging. To accommodate the complex characteristics inherent to MMW imaging modalities, we have integrated the Complex-valued Convolutional network (CCN) into the untrained learning framework for reconstruction. We took the

following steps to improve the performance of the untrained reconstruction technique in MMW imaging:

- **Network scale**: We have conducted a series of experiments to explore the optimal network scale that balances accuracy and complexity. CCN contains the fixed input and output convolution layers, as well as multiple hidden Res-blocks. Changing Res-block Numbers (RN) can vary the network scale. In general, a larger scale leads to higher accuracy, but also a longer running time, as depicted in Fig. S7 **a** and **b**. It can be observed that with fewer iterations, when RN=5, CCN achieves a balance between efficiency and reconstruction accuracy (Fig. S7 **c**). Therefore, we set the network size to RN=5.
- **Regularization**: The proposed Complex TV (CTV) regularization can achieve higher reconstruction accuracy and more stable convergence. The computational complexity of CTV is very low, so it almost has no impact on running time.
- **Quantization**: We observed that compared to traditional quantization of Floating Point 32-bit (FP32), quantization of BFloat 16-bit (BF16) can improve efficiency by 100% (Fig. S7 **f**) without affecting accuracy (Fig. S7 **e**). When using quantization with a lower bitwidth than BF16, the network may fail to converge. Therefore, we adopted BF16 quantization.

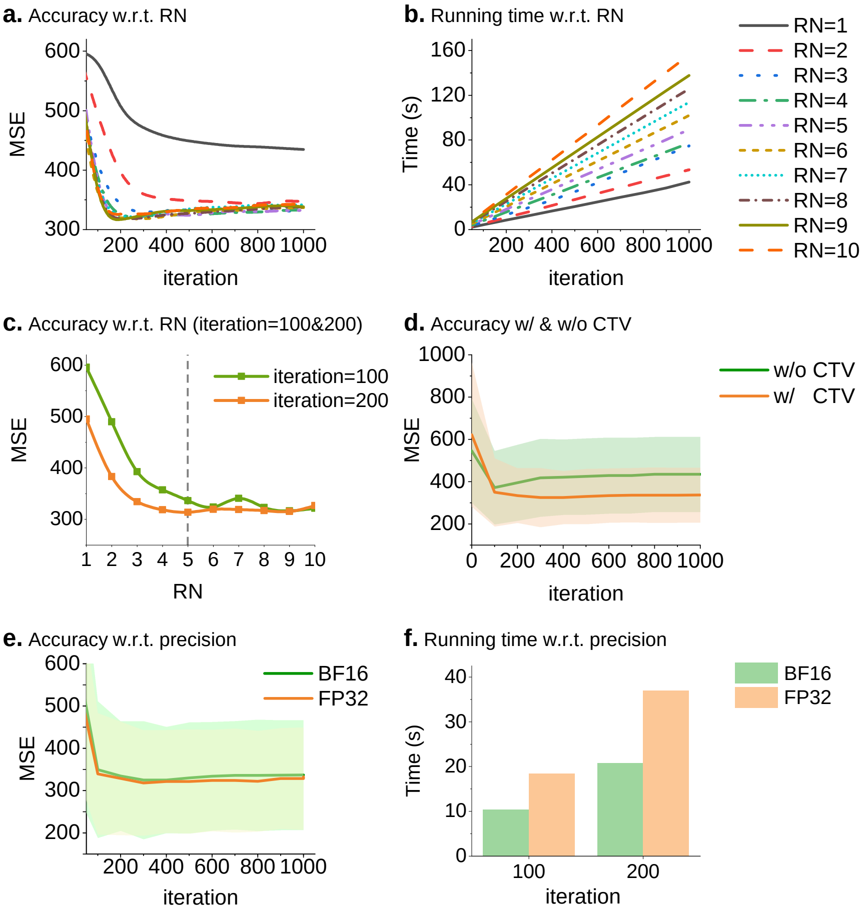

Fig. S7: Analysis of the reconstruction network. **a** and **b** show the accuracy and efficiency under different Res-block Numbers (RN). **c**, The accuracy w.r.t. RN at 100 and 200 iterations. **d**, Comparison of reconstruction accuracy with and without CTV regularization. **e** and **f** show the accuracy and efficiency of BF16 and FP32 networks.

5.3 The influence of interpolation in the scattering process

The objective of untrained learning involves the scattering process, which is denoted as

$$\mathcal{H}\left[\cdot\right] = \mathcal{F}_{2D}^{-1}\left\{ \text{IN}_k \left\{ \mathcal{F}_{3D}\left[\cdot\right] \right\} e^{-\text{j}k_y R_0} \right\}, \tag{S9}$$

where $\mathcal{F}_{3D}\left\{\cdot\right\}$ represents a 3D spatial Fourier transform for all the spatial dimensions of the imaging region. $\mathcal{F}_{2D}^{-1}\left\{\cdot\right\}$ denotes the 2D spatial inverse Fourier transform over the 2D array aperture. IN_k indicates the interpolation

with respect to the wavenumber k. To estimate the influence of interpolation, we adopt the interpolation-free scattering process which is inspired from [S85]:

$$\mathcal{H}\left[\cdot\right] = \mathcal{F}_{2D}^{-1}\left\{ \int \mathcal{F}_{2D}\left[\cdot\right] e^{-\mathrm{j}k_y(R_0+y)}\mathrm{d}y \right\}. \tag{S10}$$

The reconstruction results of the untrained learning with and without interpolation can be found in Fig. S8. Notably, the 2D and 3D images generated with or without interpolation exhibit a remarkable similarity. This similarity arises because in near-field imaging scenarios, the wavenumber variation is gradual, and interpolation is applied to slowly changing signals. In this way, simple linear interpolation can promise high-quality images. Both the imaging results and analysis have proven the robustness of the interpolation in the scattering process of the untrained learning method.

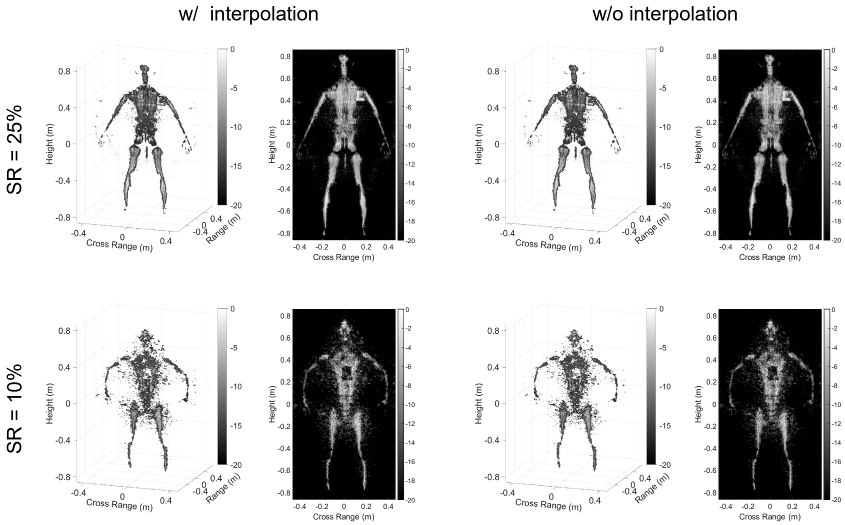

Fig. S8: Reconstructions of the untrained learning with or without interpolation in the objective.

5.4 Complexity of CCN

For convolutional neural networks, such as the one mentioned in this work, the main computational burden arises from the convolutional layers. Therefore, we will now attempt to approximate the complexity of the complex neural network by utilizing the complexity of the convolutional layers. As shown in Fig. S9, one single convolutional layer with bias has $C_iK^2HWC_o$ multiplications and $C_iK^2HWC_o$ additions, where (H, W) denotes the size of output, C_i and C_o are the channels of input x and output y, and K is the size of kernels w.

The complex convolutional layer is composed of two real-valued convolutional layers and two addition operations for output features, as illustrated in Eq. (6) in the main text. Thus, the number of Floating Point Operations (FLOPs) of a complex convolutional layer is denoted as

$$(4C_iK^2 + 2)HWC_o. \tag{S11}$$

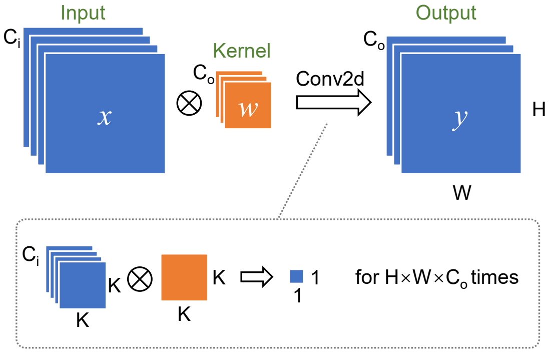

Fig. S9: The workflow of a typical convolutional layer.

When performing the gradient-based optimization for the proposed network, updating a convolutional layer requires calculating the gradient of input x_{grad} and kernels w_{grad},

$$\begin{aligned} x_{grad} &= TransConv(y_{grad}, w), \\ w_{grad} &= Conv(y_{grad}, x). \end{aligned} \tag{S12}$$

For the proposed network, the size (H, W) of output features remains unchanged. We can simplify the time complexity of computing these gradients for a complex convolutional layer as

$$\begin{aligned} x_{grad} &: (4C_oK^2 + 2)HWC_i, \\ w_{grad} &: (4C_oHW + 2)K^2C_i. \end{aligned} \tag{S13}$$

The time complexity for updating a complex convolutional layer via the gradient descent algorithm is $8C_iK^2HWC_o + 2HWC_i + 2K^2C_i$.

We note that the complexity of other operations in CCN (including ReLU, BN, and the loss function) is much lower than that of the convolutional layers. So we can approximate the total time complexity of CCN by the complexity of these convolutional layers. The proposed CCN consists of seven complex convolutional layers. The input size is $N_x \times N_z \times N_f$, where $N_x = 430$, $N_z = 186$, and $N_f = 50$. The kernel size K is 3. The number of kernels in the hidden

convolutional layers C is 128. Following Eqs. (S11, S13), we summarize the forward and backward complexity of each layer in Tab. S3. The complexity of one iteration to update CCN is the summation of all the complexities in Tab. S3. Ignoring minor terms, the total FLOPs of one iteration can be expressed as $24CK^2N_xN_zN_f + 60C^2K^2N_xN_z$.

Table S3: Time complexity of each complex convolutional layer in CCN.

Step		Complexity (FLOPs)
Forward	layer #1	$(4N_fK^2 + 2)N_xN_zC$
	layer #2-#6	$(4CK^2 + 2)N_xN_zC$
	layer #7	$(4CK^2 + 2)N_xN_zN_f$
Backward	layer #1	$8CK^2N_xN_zN_f + 2N_xN_zN_f + 2K^2N_f$
	layer #2-#6	$8C^2K^2N_xN_z + 2N_xN_zC + 2K^2C$
	layer #7	$8CK^2N_xN_zN_f + 2N_xN_zC + 2K^2C$

We summarize the complexity of all the methods we tested in the main text in Tab. S4. Compared with CS-CG, the ADMM converges with fewer iterations, thus its running time is less. Note that the complexity of the untrained method is a theoretical upper limit without any optimization techniques applied. In practical usage, we can effectively reduce the network complexity through quantization, parallel computing, and other methods, allowing the successful reconstruction within an acceptable computation time. As shown in Fig. S7 **a**, the network gradually converged between 100 to 200 iterations and took less than 21s on an NVIDIA RTX 4090 GPU.

Table S4: Comparison of the time complexity of different algorithms. N_x, N_z: Number of antennas along azimuth and height direction, respectively. N_f: Number of frequencies. N_{iter}: Number of iterations. C: Kernel number of CCN hidden layers. K: Kernel size.

Algorithm	Complexity (FLOPs)	Running time
RMA	$N_xN_zN_f\,(20 + 5\log N_xN_z + 5\log N_xN_zN_f)$	2s (2 × Intel E5-2687W)
CS-CG	$N_{\text{iter}}N_xN_zN_f(8N_f + 10\log N_xN_z)$	610s (2 × Intel E5-2687W)
ADMM	$N_{\text{iter}}N_xN_zN_f(62 + 16N_f + 20\log N_xN_z)$	66s (2 × Intel E5-2687W)
Untrained	$N_{\text{iter}}(24CK^2N_xN_zN_f + 60C^2K^2N_xN_z)$	< 21s (NVIDIA RTX 4090)

In addition, the consumer GPU RTX 4090, used for our reconstruction exhibits a significant disparity in computational power compared to professional GPUs. As illustrated in Fig. S10 **a**, the half-precision computational

power of professional GPU NVIDIA H100 is around 12 times bigger than our RTX 4090. Due to the limitations of the available devices, we do not have access to the most advanced H100 currently. Instead, we have implemented our algorithm on various GPUs including RTX 3060, RTX 2080Ti, RTX 3090, A40, and RTX 4090. Figure S10 **b** demonstrates the fact that the computational power is approximately inversely proportional to running time. On the basis of our calculations across various GPUs, this relationship can be fitted as

$$y = \frac{1544.1}{x} \quad (R^2 = 0.9889), \tag{S14}$$

where x denotes the computational power (unit: TFLOPS), and y is the running time per 100 iterations (unit: second). Using this approximate model, we estimate that the required time for H100 is less than 1.6s. The computational power of GPUs has been rapidly increasing in recent years (Fig. S10 **a**). Despite the roughness of this approximation model, we would like to emphasize that it is highly likely that a computing platform capable of high-throughput imaging, which satisfies our algorithm, is emerging in the present or near future. Therefore, we have provided a promising framework for high-throughput MMW security imaging and detection in this work.

Table S5: Running time of each step during the MMW-based security check. Here we consider the number of iterations in the reconstruction process to be 200.

GPU	Reconstruction	Detection	Total Time
RTX 4090	20.8s	9ms	21s
H100 (prediction)	1.56s	$<$ 1ms	1.7s

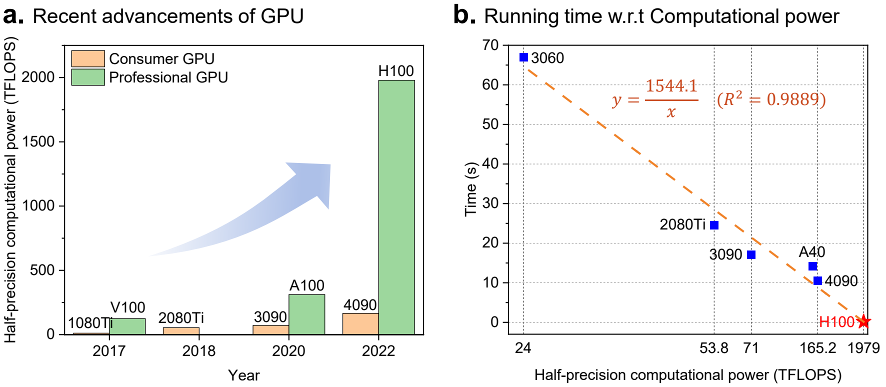

Fig. S10: The improvement in computational power facilitates the rapid calculation of our algorithm. **a**, The development in computational power of typical NVIDIA GPUs in recent years. **b**, The relationship between the running time per 100 iterations and GPU computational power. The horizontal axis is represented as the reciprocal axis. The computational time of RTX 3060, RTX 2080 Ti, RTX 3090, A40, and RTX 4090 exhibits an approximate inverse proportionality to computational power, which allows us to estimate the computation time per 100 iterations for H100 to be 0.78s.

5.5 The detection network

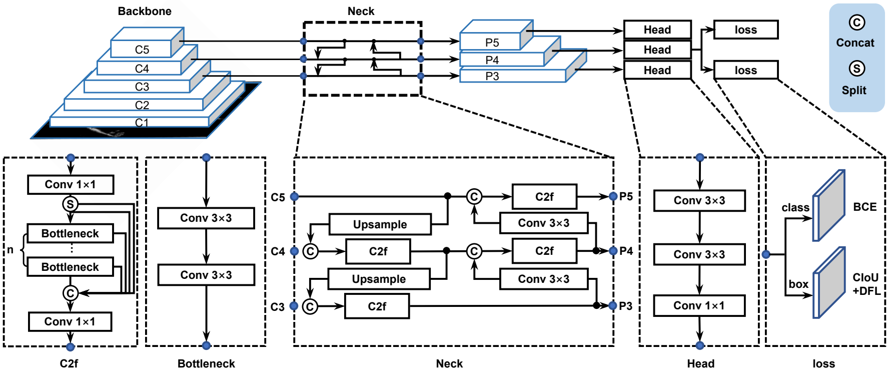

Fig. S11: The YOLOv8 network architecture.

We adopt the YOLOv8 [S86], a state-of-the-art object detection network, for post-processing detection in security inspection. Fig. S11 illustrates the network architecture, which consists of a Darknet-53 backbone [S87] with a C2f module replacing the C3 module [S88] for richer gradient flow [S86]. YOLOv8

employs a decoupled head for faster inference speed [S89] with separate classification and regression branches and no objectness branch. The classification branch uses the Binary Cross Entropy (BCE) loss [S90], while the regression branch employs the Distribution Focal Loss (DFL) [S91] and the Complete Intersection over Union (CIoU) Loss [S92]. The DFL aims to increase the probability values around the target values (i.e., y_i and y_{i+1}) by explicitly increasing the nearest category probabilities of y_i and y_{i+1} by using the formulation $S_i = \frac{y_{i+1}-y}{y_{i+1}-y_i}$ and $S_i = \frac{y-y_i}{y_{i+1}-y_i}$. The global minimum solution of DFL ensures that the estimated regression target is infinitely close to the corresponding label. The DFL is denoted as follows:

$$DFL\left(S_i, S_{i+1}\right) = -\left(y_{i+1} - y\right)\log S_i - \left(y - y_i\right)\log S_{i+1}. \tag{S15}$$

We apply the Mosaic method [S93] to augment the training dataset. Mosaic data splices four images by randomly zooming, cutting, and arranging. Following ref. [S89], the Mosaic data augmentation was employed in the first 90 epochs, followed by its removal in the last 10 epochs.

We employ commonly used metrics, including F1 score and mean Average Precision (mAP) [S94], to evaluate the quality of the detection results and assess the performance of the imaging methods. The F1 score is calculated as the harmonic mean of precision and recall, giving equal weight to both metrics. It is defined by the following formula:

$$F1 = 2\frac{Precision * Recall}{Precision + Recall}. \tag{S16}$$

In Eq. (S16), precision is defined as the ratio of correctly predicted bounding boxes to all predicted boxes, as shown below:

$$Precision = \frac{TP}{AD}, \tag{S17}$$

where TP denotes the number of correctly predicted boxes, meaning the CIoU [S94] between the predicted box and ground truth is greater than the preset threshold, and AD represents the total number of predicted boxes. The CIoU is defined as:

$$CIoU = IoU - \frac{\rho^2\left(b, b^{\mathrm{gt}}\right)}{c_0^2} - \alpha\nu, \tag{S18}$$

where IoU is the Intersection over Union, b and b^{gt} denote the predicted and ground truth boxes, respectively. ρ^2 represents the Euclidean distance between the center points of the predicted box and the ground truth, and c_0 represents the diagonal distance of the minimum closure region that can simultaneously contain both the predicted and ground truth boxes. α is denoted as $\alpha = \frac{\nu}{1-IoU+\nu}$, where $\nu = \frac{4}{\pi^2}\left(\arctan\frac{w^{\mathrm{gt}}}{h^{\mathrm{gt}}} - \arctan\frac{w}{h}\right)^2$. Here w and h denote the weight and height of any of the bounding boxes, respectively. Recall is defined

as the ratio of correctly matched ground truth boxes to all ground truth boxes and is expressed as:

$$Recall = \frac{TP}{AT}, \tag{S19}$$

where AT represents the total number of ground truth boxes.

The Average Precision (AP) is calculated as the area under the Precision-Recall curve for each query. The mAP is calculated by averaging APs across all queries, considering both precision and recall of an algorithm, thus providing a more comprehensive evaluation. The mAP50 indicates the mean average precision when the CIoU is greater than 0.5, and mAP50-95 refers to the mean value of mAP for CIoU starting from 0.5 to 0.95 with an interval of 0.05.

In the experiments, we first selected 1200 full-sampled echo reconstructions by RMA, 1000 used for training and 200 for testing. The dataset includes concealed objects such as knife, wrench, phone, gun, and explosive powdered material (using silica gel instead in experiments) carried by the human body. The weighting coefficients for the box loss, classification loss, and DFL are 7.5, 0.5, and 1.5, respectively. The training process comprises 100 epochs to ensure convergence. We adopt the polynomial decay learning rate scheduling strategy with an initial learning rate of 0.1, while the momentum and weight decay are set as 0.937 and 0.0005, respectively. The training was implemented on an NVIDIA RTX 3090 GPU with a batch size of 32. After convergence, we applied the detection network for reconstructed images by RMA, CS-CG, ADMM, DL, and the proposed untrained learning under both random and statistically sparse sampling. As illustrated in Tab. S6 and Tab. S7, we can draw the following conclusions:

- The statistically optimized arrays outperform the random arrays in detection accuracy, indicating higher reconstruction quality.
- The untrained learning technique outperforms all the other reconstruction methods in detection accuracy, validating its effectiveness.
- The combination of statistically sparse array and untrained learning leads to the best detection performance among all the combinations of sparse strategies and reconstruction methods.

To adapt images of sparse sampling, we reconstructed all training and test set images under the proposed statistical sparse sampling. We trained the detection networks using reconstructions with sampling ratios of 10% and 25%, respectively. The visual demonstration of detection results is shown in Fig. S12. The numerical results are shown in Tab. S8. These networks exhibit higher accuracy compared to the networks trained only on full sampling reconstructions.

Table S6: Comparison of detection indicators for results of different sampling strategies and reconstruction methods at 25% sampling ratio. The detection network is trained with full-sampled echo reconstructions.

Array	Full	Random					**Statistically optimized**				
Methods	RMA	RMA	CS-CG	ADMM	DL	**Untrained**	RMA	CS-CG	ADMM	DL	**Untrained**
F1	0.7710	0.1088	0.0120	0.0023	0.1804	**0.2853**	0.3731	0.2270	0.0559	0.3866	**0.5903**
mAP50	0.7720	0.0733	0.0143	0.0008	0.1210	**0.2490**	0.3340	0.1840	0.0368	0.3110	**0.5570**
mAP50-95	0.4590	0.0354	0.0064	0.0004	0.0649	**0.1420**	0.1770	0.0873	0.0147	0.1860	**0.3180**

Table S7: Comparison of detection indicators for results of different sampling strategies and reconstruction methods at 10% sampling ratio. The detection network is trained with full-sampled echo reconstructions.

Array	Full	Random					**Statistically optimized**				
Methods	RMA	RMA	CS-CG	ADMM	DL	**Untrained**	RMA	CS-CG	ADMM	DL	**Untrained**
F1	0.7710	0.0000	0.0000	0.0000	0.0000	**0.0473**	0.0167	0.0000	0.0000	0.1041	**0.1668**
mAP50	0.7720	0.0000	0.0000	0.0000	0.0000	**0.0396**	0.0166	0.0000	0.0000	0.0816	**0.1260**
mAP50-95	0.4590	0.0000	0.0000	0.0000	0.0000	**0.0169**	0.0100	0.0000	0.0000	0.0360	**0.0745**

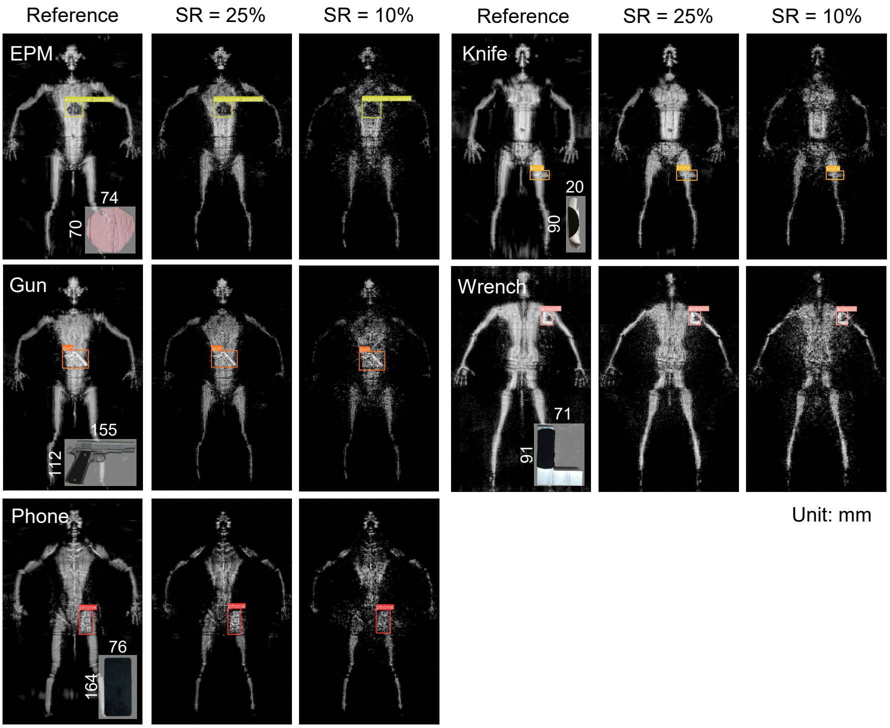

Fig. S12: Reconstructed images and detection results in cases of different target objects including explosive powdered material, gun, phone, knife, and wrench.

Table S8: Detection results in cases where the detection network is trained with reconstructions of statistically sparse echoes.

Sampling ratio	10%	25%
F1	0.4811	0.6180
mAP50	0.4380	0.6480
mAP50-95	0.2300	0.3550

6 More details about the transmit and receive antenna isolation and the calibration procedure

The element spacing between two adjacent transmit (receive) antennas in our system is 10mm, while the spacing between the nearest transmit and receive antennas is 18.9mm. In general, when the spacing between two adjacent antennas falls below half of the wavelength, the isolation between them tends to deteriorate, potentially leading to a significant coupling effect. In the context of the detection system illustrated in Fig. 5 of the main text, the working frequency's wavelength is approximately 10mm, which is nearly half of the spacing between adjacent transmit and receive antennas. Consequently, the coupling effect among elements is not pronounced, and we can assert that the transmit and receive isolation is adequately maintained, which is practically 40 dB.

Furthermore, to reduce the impact of coupling among elements and address channel inconsistencies, we employ the following calibration methods:

1. Placement of a Metal Plate: A metal plate is positioned in parallel to the antenna array.

2. Theoretical Reference Echo: We establish the theoretical reference echo of the metal plate as

$$S_{\text{theory}}(k, y) = e^{-\mathrm{j}kr}, \tag{S20}$$

where k denotes the wavenumber, and r signifies the distance between the metal plate and the antenna array.

3. Calibration Factor: The calibration factor is computed as

$$S_{\text{cali}}(k, y) = \frac{S_{\text{theory}}(k, y)}{S_{\text{metal}}(k, z)}, \tag{S21}$$

where $S_{\text{metal}}(k, z)$ represents the echo obtained from the metal plate.

4. Echo Calibration in Application: For practical echo calibration, we employ the following equation:

$$S_{\text{calied}}(k, y) = [S_{\text{mea}}(k, y) - S_{\text{air}}(k)] \cdot S_{\text{cali}}(k, y), \tag{S22}$$

where $S_{\text{mea}}(k, y)$ denotes the measured echo, and $S_{\text{air}}(k)$ corresponds to the echo generated in the air.

By following this calibration procedure, we effectively minimize the coupling effects among the transmit and receive antennas while simultaneously reducing channel inconsistencies.

7 More comparisons on the test dataset

7.1 Additional reconstruction results on the test dataset

We tested the performance of the proposed method on the test set under various sampling ratios. As mentioned in the main text, the test set consists of 200 randomly selected MMW echo signals. We compared the proposed untrained learning method with RMA, CS-CG, ADMM, and deep learning (DL) techniques. The visual and numerical comparisons are shown in Fig. S13 and Fig. S14, respectively. We can conclude that:

- The statistically sparse array outperforms the randomly sparse array.
- The deep learning method, while performing well in certain scenarios, lacks robustness and may perform worse in certain cases.
- The untrained learning method outperforms the other comparative methods in cases of average reconstruction accuracy and robustness.

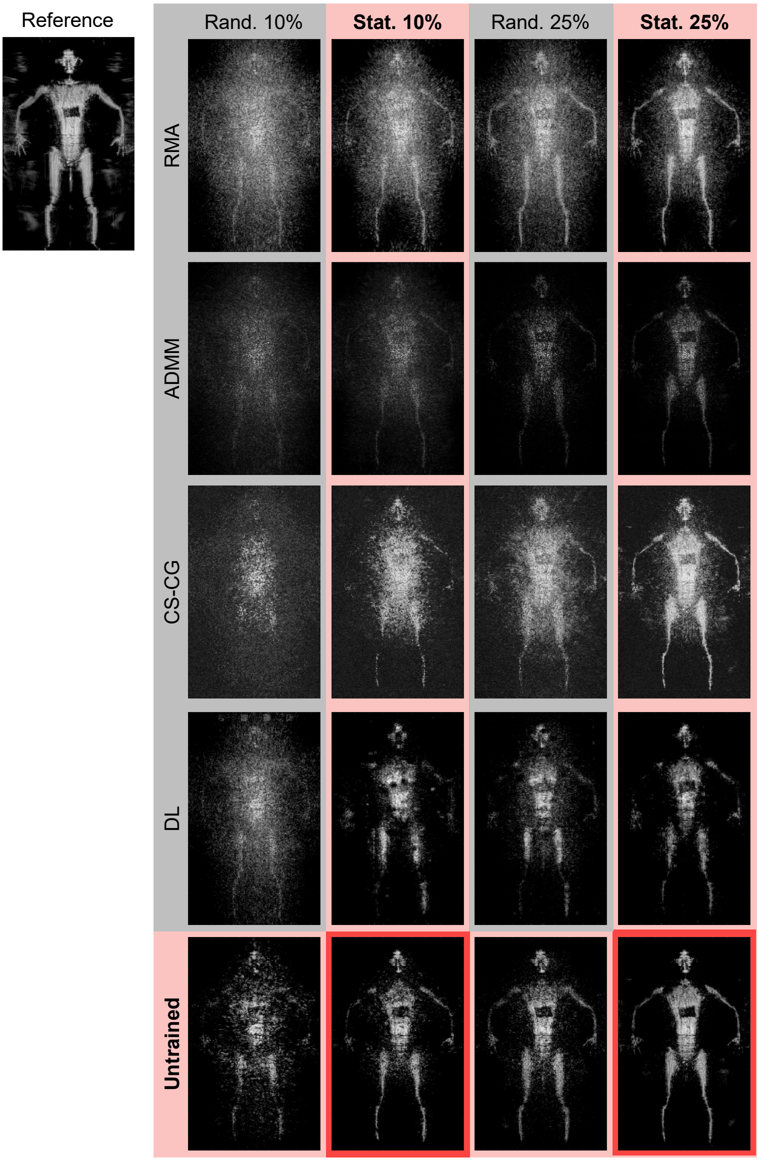

Fig. S13: Visual comparisons of various algorithms. We tested the reconstructions from both random and statistically optimized arrays at 10% and 25% sampling ratios.

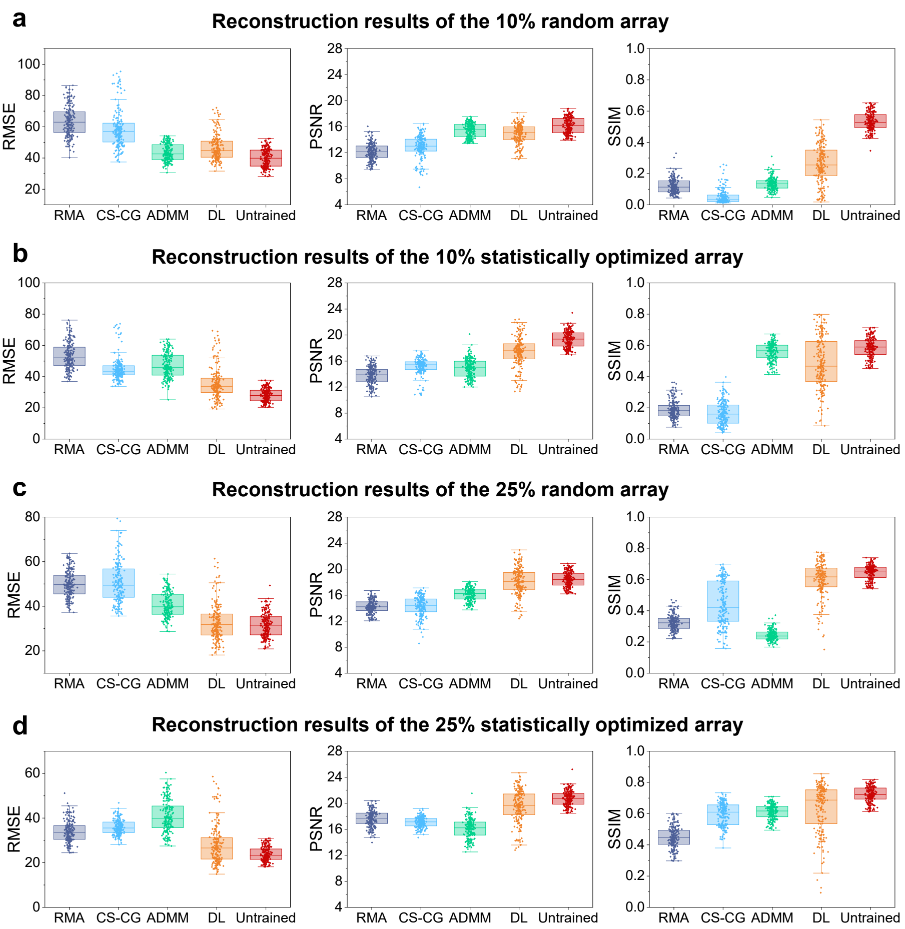

Fig. S14: The numerical comparisons between different reconstruction techniques including RMA, CS-CG, ADMM, DL, and the proposed untrained learning. The test dataset contains 200 different echoes. The comparisons involve the evaluated RMSE(↓), PSNR(↑), and SSIM(↑) values of 10% randomly sampled (**a**), 10% statistically sparse sampled (**b**), 25% randomly sampled (**c**), and 25% statistically sparse sampled (**d**) echoes.

7.2 Additional comparisons among different sparse arrays

This section conducts a comparative analysis between the proposed statistically optimized sparse array and other prevalent sparse array configurations, including both random and regular topologies. The random sparse array is generated through direct random sampling from a full array scheme [S38]. Random arrays can introduce sidelobes, leading to degraded reconstruction quality.

The regular array is characterized by uniform element spacing. One crucial factor is that uniform element spacing, when not aligned with the Nyquist sampling criterion, often leads to the emergence of grating lobe artifacts within the reconstructed images. These artifacts manifest as ghost targets and can potentially compromise the overall image quality. This phenomenon is particularly evident when the element interval restricts the imaging region, leading to the presence of grating lobes. The azimuth and height arrays encompass transmitting and receiving apertures, respectively, and their unambiguous distance in the azimuth direction is influenced by the array's design parameters.

As articulated in ref. [S11], when $\frac{\lambda_c}{\Delta_x} \leq \Theta$, the azimuth unambiguous distance D_x is given by the formula:

$$D_x = \frac{\lambda_c \cdot R_0}{\Delta_x}, \tag{S23}$$

where D_x represents the azimuth unambiguous distance, λ_c signifies the wavelength of the center operating frequency, R_0 pertains to the imaging distance, Δ_x denotes the element interval, and Θ corresponds to the antenna's beamwidth. Conversely, when $\frac{\lambda_c}{\Delta_x} > \Theta$, the presence of ghost targets becomes less conspicuous; however, it comes at the cost of reduced azimuth resolution. The manifestation of ghost targets is linked to the relationship between λ_c and Δ_x relative to the antenna beamwidth Θ.

Considering the parameters used in our experiments, the azimuth unambiguous distance, D_x, exceeds 0.75 meters. This is sufficient for effective security checks in near-field imaging scenarios. However, when half of the azimuth elements are removed (sampling ratio of 25%), reducing the element spacing, D_x drops to 0.375 meters. This reduction signifies a severe aliasing phenomenon, where ghost effects become prominent. Therefore, the ambiguity phenomenon is intricately linked to the specific parameter settings.

Furthermore, we conducted a series of experiments to compare different arrays (random, regular, and statistically optimized) with various reconstruction algorithms, including RMA, CS-CG, ADMM, DL, and untrained learning. The evaluations on the test dataset (200 echoes in total) in Tab. S9 and Fig. S15 indicate that statistically optimized arrays outperform random and regular arrays across all reconstruction algorithms. Additionally, visual comparisons in Fig. S16 and Fig. S17 validate the aforementioned analysis, indicating that random arrays tend to introduce high levels of noise, while regular arrays tend to produce ghost artifacts. Regular arrays introduce grating lobes, which interfere with primary body regions, resulting in image distortion and yielding inferior metrics in most cases. The statistically optimized arrays achieve a delicate balance between noise reduction and minimizing grating lobes, thus outperforming the random and regular arrays we tested.

Table S9: The quantitative comparisons of reconstructed data with different sampling strategies (regular, random, and statistically optimized) and reconstruction methods (RMA, CS-CG, ADMM, DL, and Untrained). We used all the 200 echoes of the test dataset for reconstruction. The values specified to the right of the forward slash indicate the variances.

Method	SR	10%			25%		
	Metrics	RMSE	PSNR	SSIM	RMSE	PSNR	SSIM
RMA	Regular	67.05/8.13	11.67/1.06	0.15/0.07	65.83/6.27	11.80/0.84	0.27/0.07
	Random	63.66/9.63	12.13/1.32	0.12/0.05	53.33/8.49	13.75/1.36	0.19/0.06
	**Statistically optimized**	**49.92/5.97**	**14.26/1.00**	**0.32/0.05**	**33.89/4.86**	**17.65/1.25**	**0.45/0.07**
CS-CG	Regular	67.85/16.07	11.73/1.96	0.03/0.011	66.70/9.54	11.73/1.20	0.06/0.02
	Random	59.10/13.49	12.91/1.72	0.05/0.05	44.90/7.39	15.22/1.25	0.16/0.07
	**Statistically optimized**	**50.93/9.75**	**14.28/1.52**	**0.44/0.14**	**35.94/3.28**	**17.07/0.77**	**0.60/0.07**
ADMM	Regular	46.09/6.02	14.93/1.13	0.12/0.04	47.65/7.83	14.69/1.44	0.46/0.07
	Random	43.58/5.65	15.41/1.09	0.13/0.04	47.15/7.96	14.90/1.45	0.56/0.06
	**Statistically optimized**	**40.82/5.67**	**16.11/1.00**	**0.24/0.03**	**40.91/7.22**	**16.09/1.54**	**0.61/0.05**
DL	Regular	67.05/14.72	11.80/1.84	0.16/0.16	59.65/12.96	12.81/1.79	0.17/0.19
	Random	46.62/8.23	14.88/1.45	0.26/0.13	35.65/10.09	17.40/2.28	0.49/0.16
	**Statistically optimized**	**32.76/8.11**	**18.07/2.05**	**0.60/0.11**	**28.05/8.86**	**19.55/2.50**	**0.64/0.15**
**Untrained**	Regular	50.84/6.22	14.07/1.07	0.47/0.09	45.65/6.15	15.02/1.13	0.46/0.09
	Random	40.05/5.84	16.17/1.23	0.53/0.06	28.23/4.20	19.30/1.24	0.59/0.06
	**Statistically optimized**	**31.53/5.42**	**18.40/1.16**	**0.65/0.05**	**23.90/3.31**	**20.72/1.15**	**0.72/0.05**

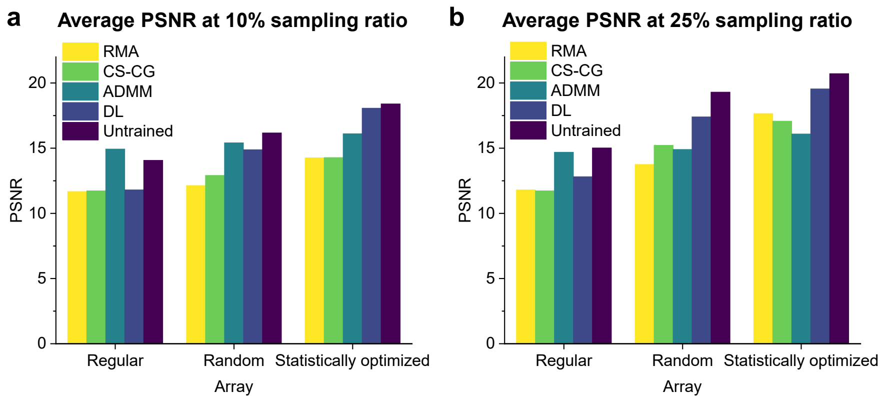

Fig. S15: Average PSNR of reconstructed data with different sampling strategies (regular, random, and statistically optimized) and reconstruction methods (RMA, CS-CG, ADMM, DL, and Untrained) on the test dataset. **a**, Average PSNR at 10% sampling ratio. **b**, Average PSNR at 25% sampling ratio. All the reconstruction methods obtained the best average PSNR on the statistically optimized array and the worst average PSNR on the regular array at both 10% and 25% sampling ratios.

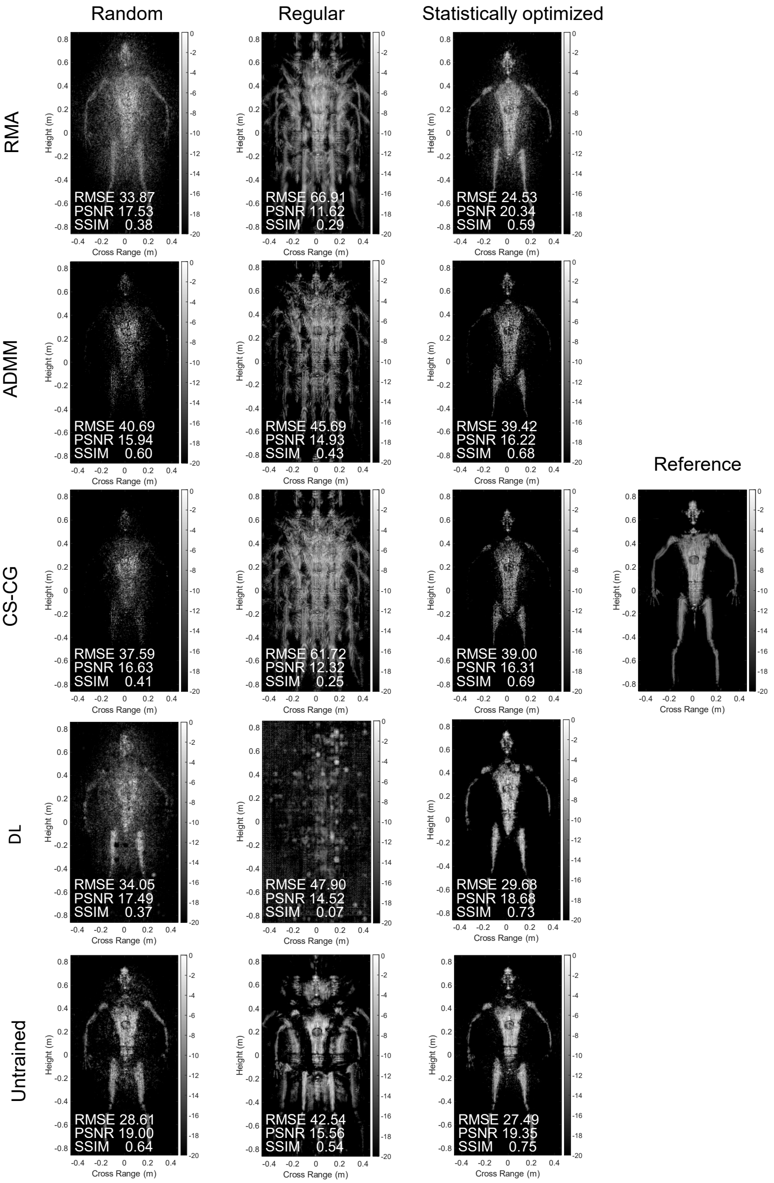

Fig. S16: Reconstruction results by RMA, ADMM, CS-CG, DL, and the untrained learning technique under 25% sampling ratio of different sparse arrays including random, regular, and statistically optimized arrays. The reconstructions of the regular sparse array contain severe artifacts. The reported statistically optimized array achieves superior reconstruction performance compared to random and regular arrays.

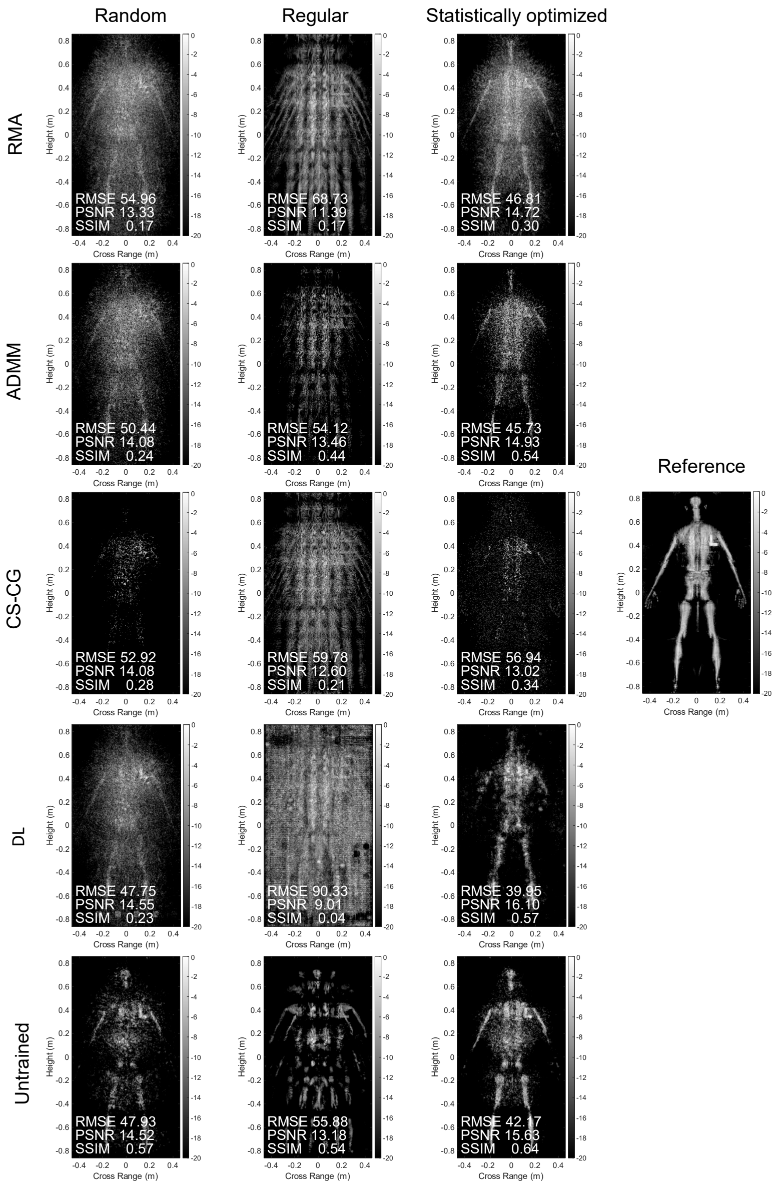

Fig. S17: Reconstruction results by RMA, ADMM, CS-CG, DL, and the untrained learning technique under 10% sampling ratio of different sparse arrays including random, regular, and statistically optimized arrays. The reconstructions of the regular sparse array contain severe artifacts. The reported statistically optimized array achieves superior reconstruction performance compared to random and regular arrays.

8 More discussions about applicability and generalization

We conducted a series of experiments to reveal the applicability and generalization the reported system. The experiments involved clothing, body shape, subject position, target position and status, and element assembly error. The reference images are reconstructed from full-sampled echoes by RMA, while the sparsely sampled images are reconstructed by the untrained method. We evaluated the RMSE, PSNR, and SSIM values of under-sampled reconstructions with the above-mentioned references as benchmarks.

8.1 Clothing material and thickness

As shown in Fig. S18, we can detect hidden objects under cotton, synthetic fiber, and blended fabric clothes. While other materials exhibit good reconstruction quality and facilitate concealed object identification, leather impedes MMW, making it difficult to detect hidden targets. Wool, on the other hand, is somewhat MMW-penetrable. However, it interferes with the detection of MMW-absorbing materials such as EPM. So we recommend operators request the subject take off wool, leather, and fur clothing when performing MMW security checking. Further, we tested the reconstruction and detection results in cases of various clothing thicknesses, as shown in Fig. S19. For MMW-penetrated clothing materials, it does not affect the reconstructed image quality and detection accuracy whether the subject is wearing a single jacket or the common layering of multiple thick garments (Down jacket + sweater + T-shirt).

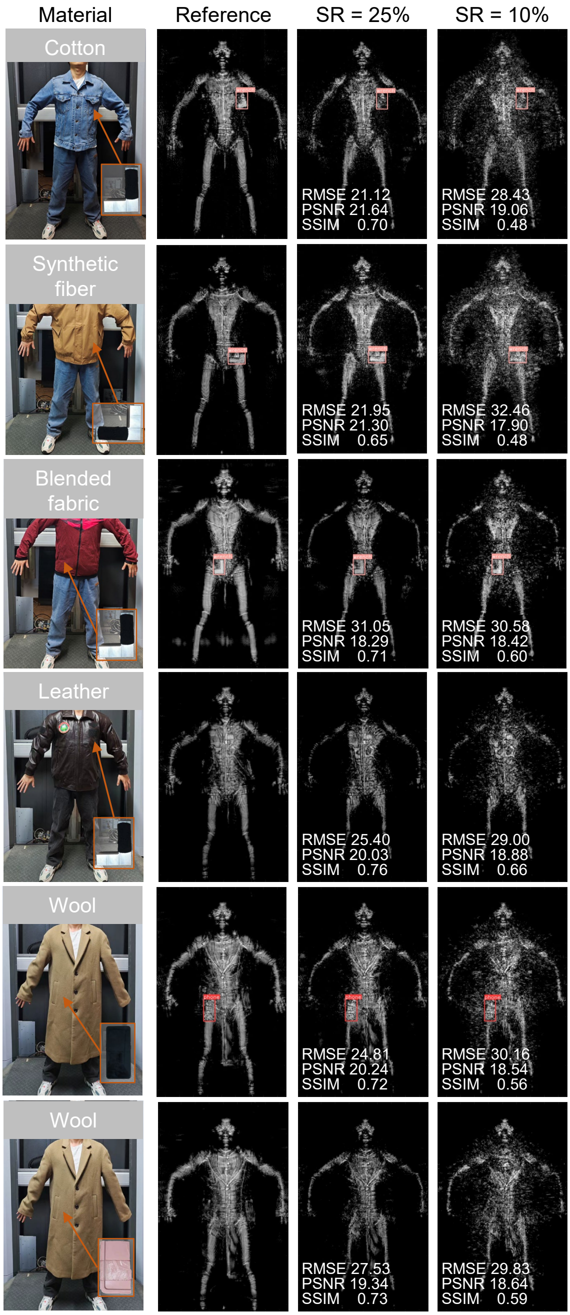

Fig. S18: Reconstructed images and detection results with different types of clothing material. The leather cloth impedes MMW, resulting in the inability or misleading to identify hidden targets. The wool cloth may affect the detection of MMW-absorbing targets such as EPM.

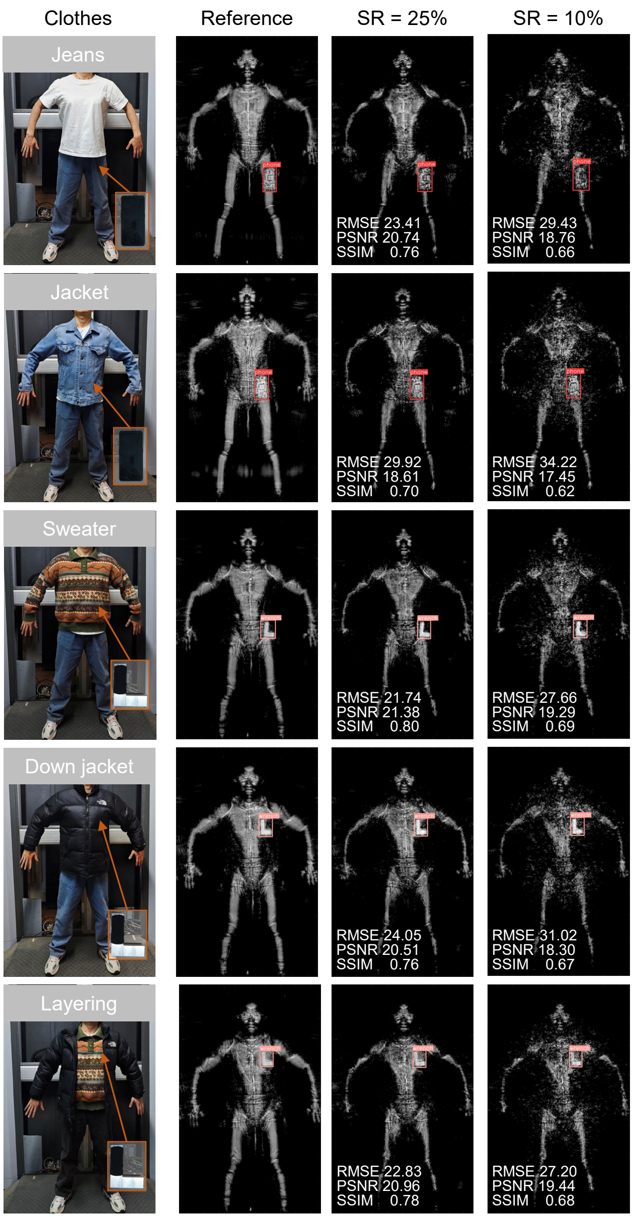

Fig. S19: Reconstructed images and detection results with different clothing thicknesses. The proposed scheme is not sensitive to the thicknesses of MMW-penetrable clothes.

8.2 Body shape

We tested several cases, including heights ranging from 160cm to 189cm and weights ranging from 45kg to 95kg. The reconstruction and detection results are shown in Fig. S20. The imaging coverage area of our system is set at around 185cm in height and 90cm in width, which can be validated by the reconstruction results. Even in the case where subjects have a height exceeding 185cm, the target hidden in the clothes can also be detected. The proposed system can successfully image and detect the concealed targets of different people with common body shapes. We aim to gather a broader dataset that encompasses a greater variety of body shapes to further promote the applicability of the proposed system across a wider range of real-world use cases.

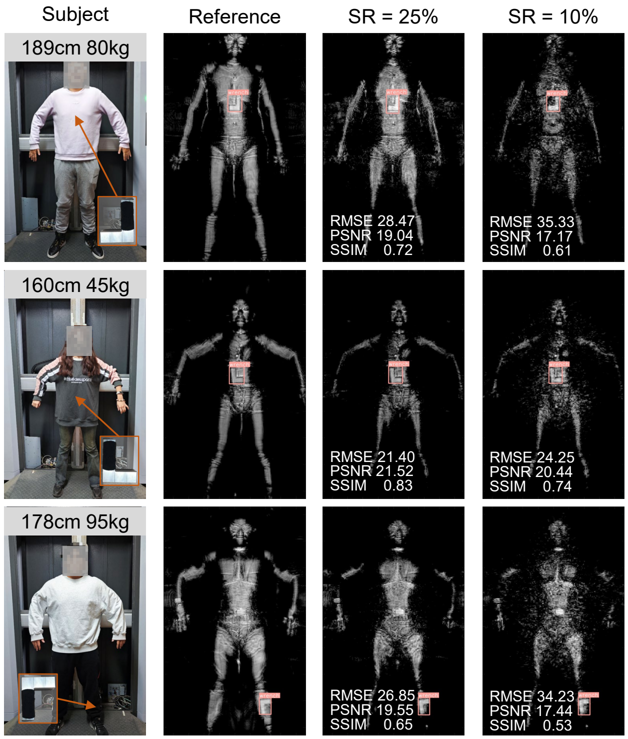

Fig. S20: Reconstructed images and detection results in cases of various body shapes with heights ranging from 160cm to 189cm and weights ranging from 45kg to 95kg. The proposed scheme can deal with subjects with various body shapes.

8.3 Subject position

We conducted a series of experiments to reveal the performance boundary in cases where the subject is positioned off-center or rotates away from the central position. The feasible range for left and right movement of the subject is ±40cm, and the range for forward and backward movement is ±20cm. Besides, we tested situations with the subject facing forward (0°) and at angular deviations of 10°, 20°, 30°, and 45°. The numerical evaluations of offset forward/backward/to the left/to the right are shown in Fig. S21. The reconstruction and detection results are shown in Fig. S22 (offset to the right), Fig. S23 (offset forward & backward), and Fig. S24 (subject rotation). We can draw the main conclusions as follows:

- In the context of PSNR, the image quality reconstructed by the random and statistically optimized arrays will decrease when the subject deviates from the central position Fig. S21. In general, the larger the deviation, the lower the quality.
- However, a few examples may not follow the above rules. This is mainly because the bigger the deviation, the interference of the random array would also deviate, leading to greater sidelobe separation from the primary body. Consequently, some portions of the subject may appear dim, and PSNR may become better.
- SSIM typically decreases as the subject deviates from the central position. In the scenarios of the subject moving right and backward, SSIM initially decreases and then increases, as shown in Fig. S22 and Fig. S23. This is because when the deviation exceeds a certain range, the expansion of the black areas in the image without the human body may lead to an increase in SSIM.
- As shown in Fig. S22 and Fig. S23, when the subject deviates from the central position, the detectable range is:
  - 25% sampling ratio: forward 10cm, backward 20cm, left and right 40cm;
  - 10% sampling ratio: forward 10cm, backward 20cm, left and right 15cm.
- When the subject's body rotates, the detectable range is within 20° for both 25% and 10% sampling ratios (Fig. S24). In actual use, when it is greater than 20°, the body will show an obvious rotation, and the operator should promptly remind the person being tested to adjust their posture.

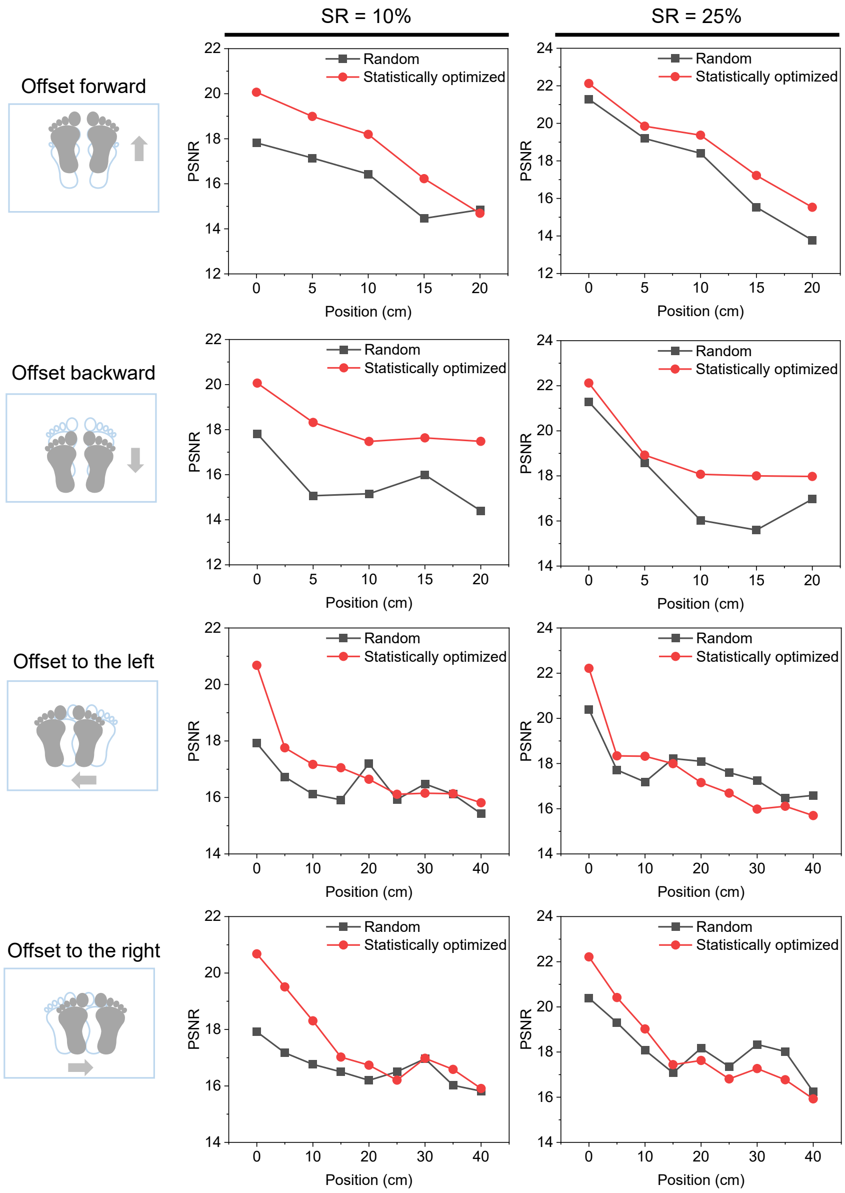

Fig. S21: Reconstruction results of the random and statistically optimized sparse arrays in cases where the subject being tested is offset from the central position for inspection. The case of the subject standing at the central position was treated as the benchmark.

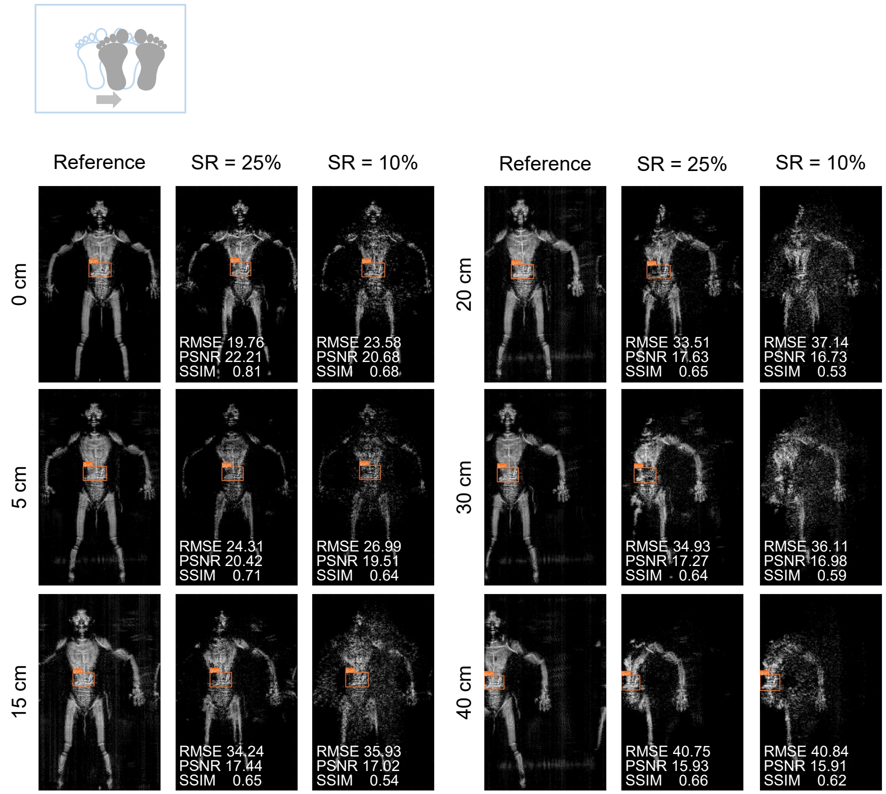

Fig. S22: Reconstructed images and detection results in cases where the subject is offset to the right from the central position. The case of the subject standing at the central position was treated as the benchmark.

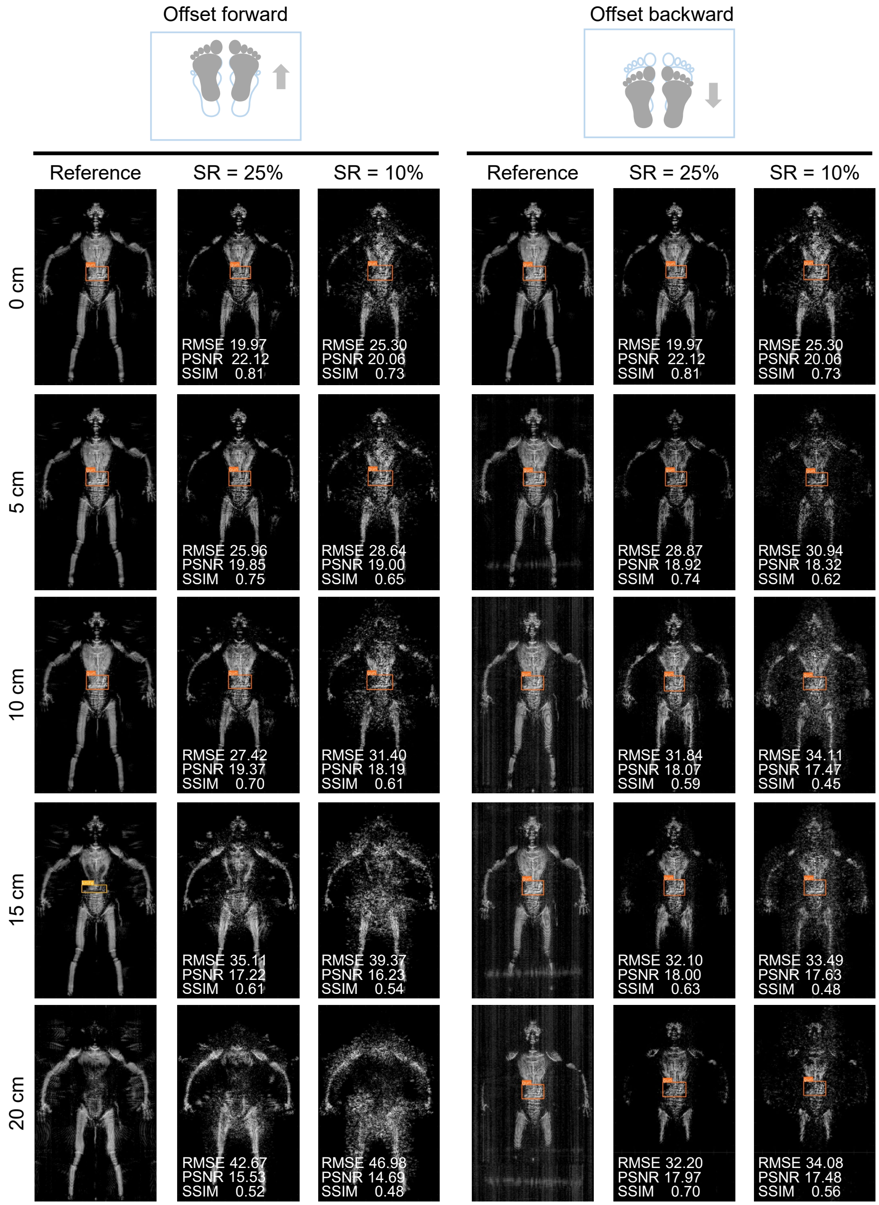

Fig. S23: Reconstructed images and detection results in cases where the subject is offset forward or backward from the central position. The case of the subject standing at the central position was treated as the benchmark.

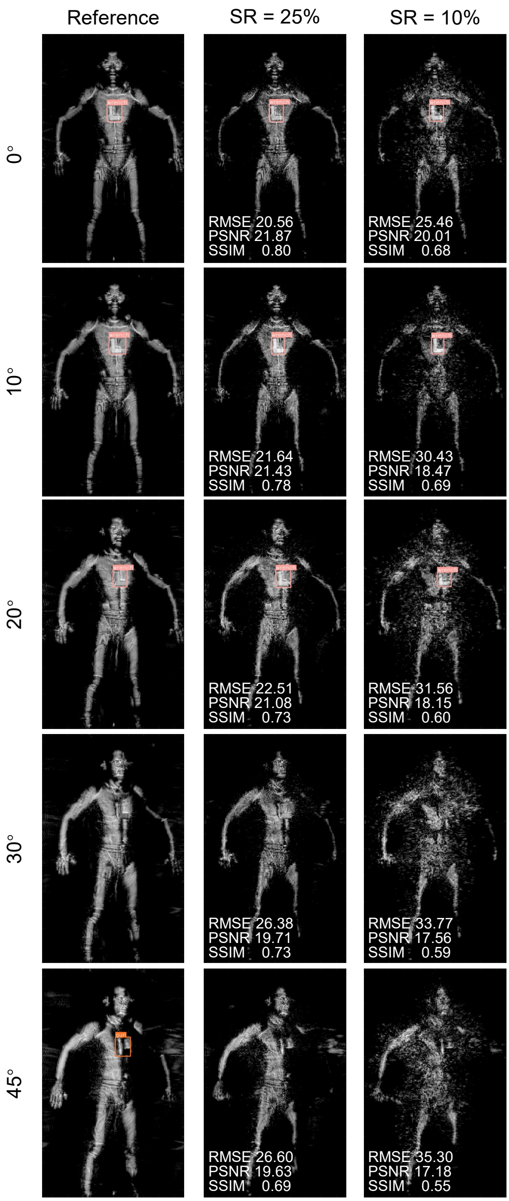

Fig. S24: Reconstructed images and detection results in cases where the subject faces different directions. We tested situations with the subject facing forward (0°) and at angular deviations of 10°, 20°, 30°, and 45°.

8.4 Target position and status

Generally, the concealed targets are relatively small compared to the human body. Thus, the variations in targets' positions and statuses lead to a relatively minor influence in RMSE, PSNR, and SSIM compared to the variations in the subject's posture and position. As for detection, the utilized YOLO network has demonstrated its effectiveness in handling variations in targets' positions and statuses across natural images [S86]. When we migrate it to the MMW image scenario, the network also exhibits robustness to these variations. As shown in Fig. S26, the object can be successfully detected when hidden within the detectable range of the human body. The detection network is robust to changes in object rotation and open-closed status of knives, as shown in Fig. S25.

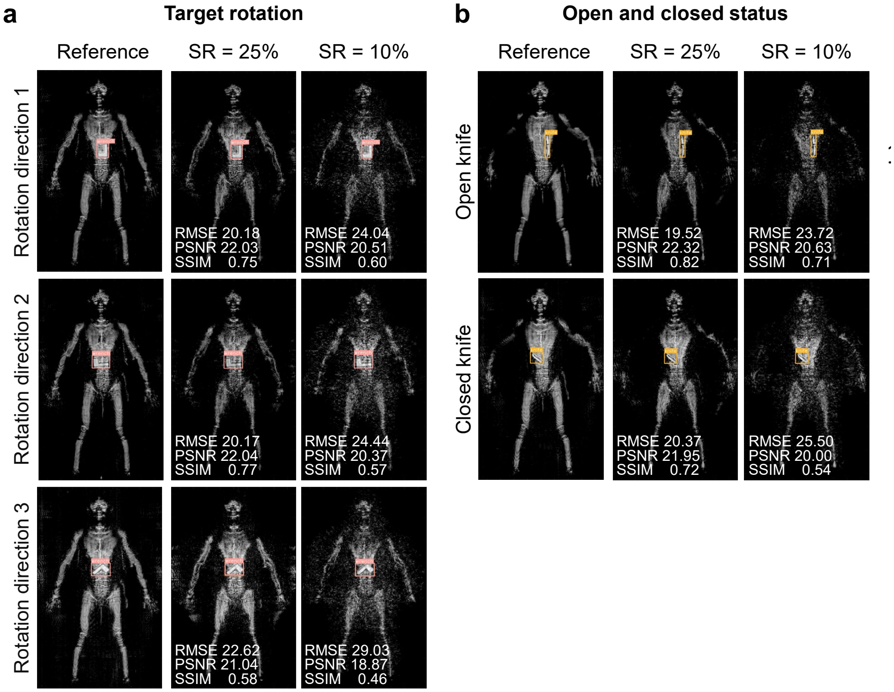

Fig. S25: Reconstructed images and detection results in cases of different object orientations and open-closed statuses. The detection network is robust to changes in the rotation and open-closed status of objects.

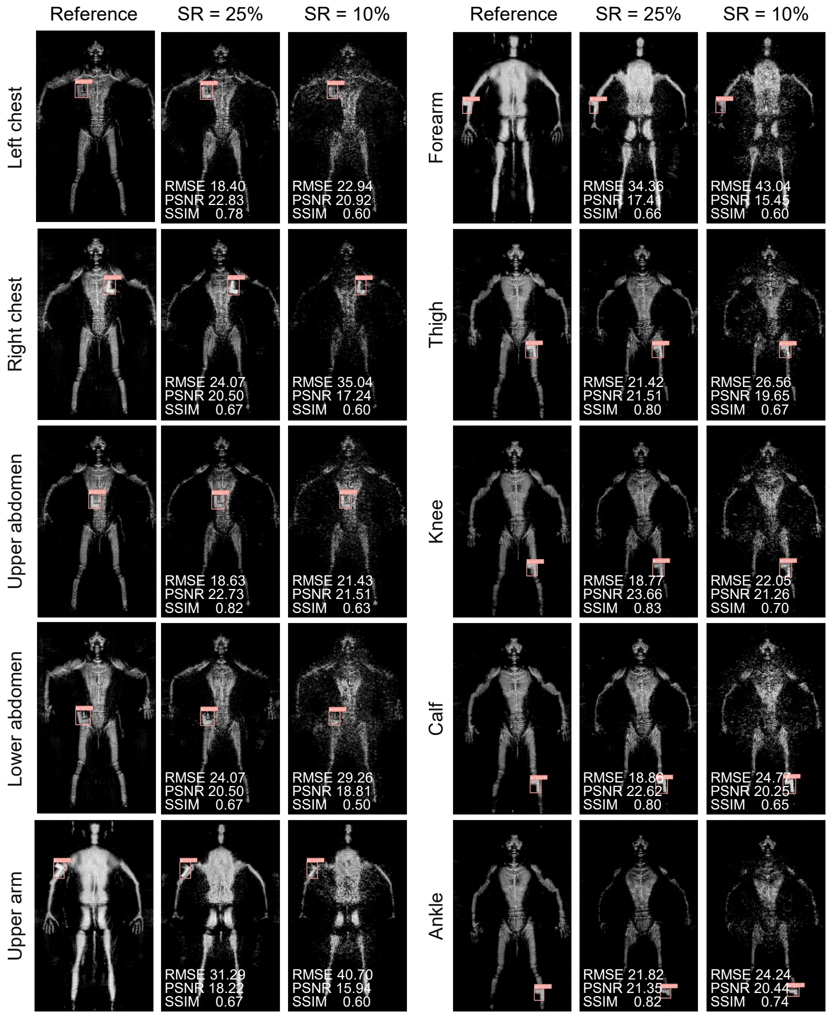

Fig. S26: Reconstructed images and detection results in cases of different object positions. The detection network is robust to changes in object positions.

8.5 Assembly position error of array elements

To investigate the error in element positioning, we have employed full-wave electromagnetic simulation utilizing FEKO software [S95]. The imaging target is a resolution board with some hollow stripes. We have configured a square array, the parameters of which are detailed in Tab. S10. The original element spacing of the full array (D) is 5mm. We introduced a random deviation to the array element positions, characterized by a standard deviation $V \times D$.

We tested both the full array and sparse array (sampling ratio is 25%), as shown in Fig. S27. The reconstructed images by untrained learning are shown in Fig. S28, while presuming that the elements are correctly positioned. We also present the reference imaging results of the full arrays by RMA under the same element position errors.

The imaging results obtained through the proposed untrained learning method exhibit gradual and slight distortion as the element position error increases. Specifically, the amplitude exhibits minor fluctuations when V reaches 50%. Remarkably, under the same element position error, the imaging results achieved through the proposed untrained learning method closely resemble those produced by full arrays. Even if $V = 100\%$, we can still distinguish the texture and structure of the target. In contrast, the other methods applied to the same sparse arrays yield distorted images. Consequently, we can conclude that the proposed untrained learning method exhibits robustness in the face of varying element position errors.

Table S10: FEKO simulation parameters for 2D sparse array

Parameters	Values
Imaging distance (R_0)	0.6 m
Start frequency	30 GHz
Stop frequency	35 GHz
Number of frequency steps	51
Number of antennas of the full array	128 × 128
Element spacing of the original full array (D)	5 mm
Sampling ratio of the sparse array	25%
Azimuth/Height resolution	5 mm
Range resolution	30 mm
Standard deviation of element position error	$V \times D$, $V = 0\% \rightarrow 100\%$

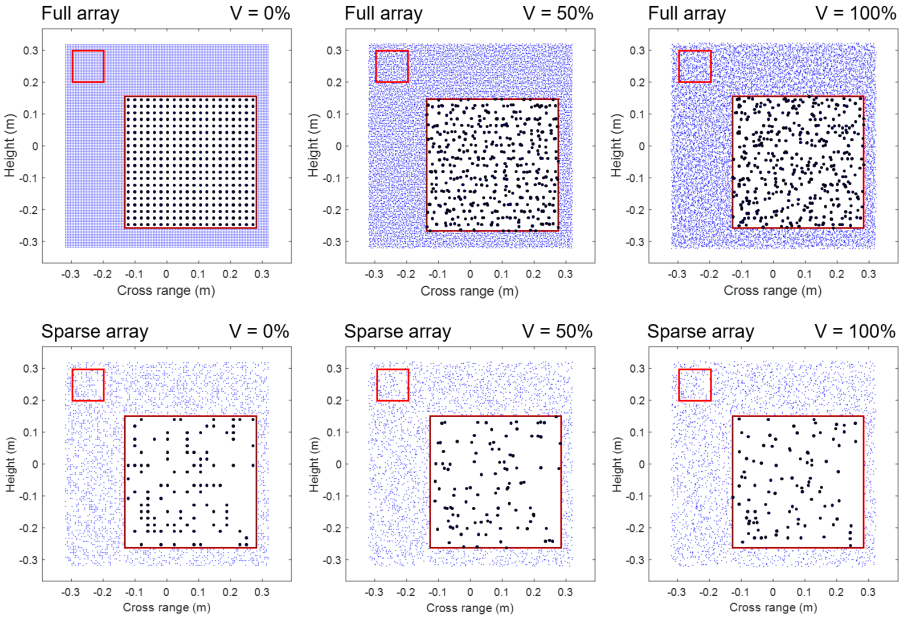

Fig. S27: FEKO simulation arrays of various element position variance ratios (V = 0%, 50%, and 100%) for the full array (first row) and the sparse array (second row).

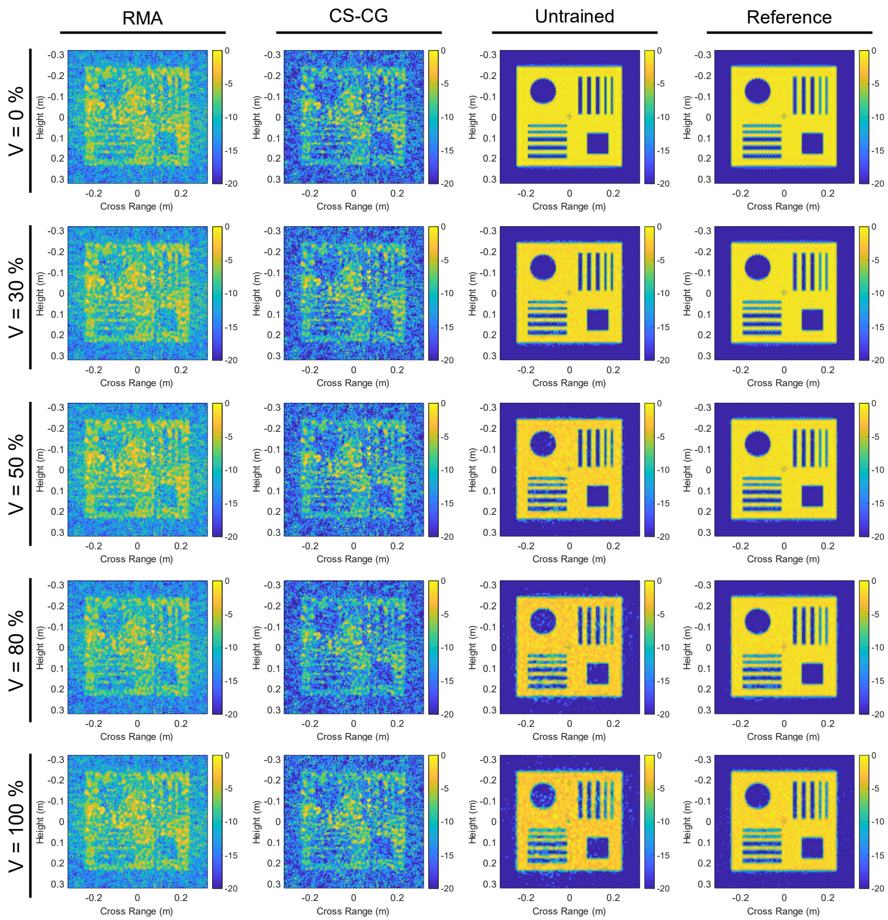

Fig. S28: Reconstructions of the FEKO simulations with diverse element position variance ratios. The first, second, and third columns correspond to the reconstructed images of RMA, CS-CG, and untrained learning under a 25% sparsely sampling ratio. The fourth column (reference) images are reconstructed by RMA from full-sampled echoes. The element position variance ratios (V) correspond to 0%, 30%, 50%, 80%, and 100%. The proposed untrained learning method stands as the most robust approach for mitigating element position error among the aforementioned techniques.

References

[S1] Sheen, D.M., McMakin, D.L., Collins, H.D., Hall, T.E.: Near-field millimeter-wave imaging for weapons detection. In: Appl. Signal Image Process. Explosives Detection Syst., vol. 1824, pp. 223–233 (1993). SPIE

[S2] Sheen, D.M., McMakin, D.L., Hall, T.E.: Three-dimensional millimeter-wave imaging for concealed weapon detection. IEEE Trans. Microw. Theory Techn. **49**(9), 1581–1592 (2001)

[S3] Keller, P.E., McMakin, D.L., Sheen, D.M., McKinnon, A.D., Summet, J.W.: Privacy algorithm for airport passenger screening portal. In: Appl. Sci. Computat. Intell. III, vol. 4055, pp. 476–483 (2000). SPIE

[S4] Keller, P.E., McMakin, D.L., Sheen, D.M., McKinnon, A.D., Summet, J.W.: Privacy algorithm for cylindrical holographic weapons surveillance system. IEEE Aerosp. Electron. Syst. Mag. **15**(2), 17–24 (2000)

[S5] McMakin, D.L., Sheen, D.M., Hall, T.E.: Millimeter-wave imaging for concealed weapon detection. In: Nondestruct. Detection Meas. Homeland Secur., vol. 5048, pp. 52–62 (2003). SPIE

[S6] Sheen, D.M., McMakin, D.L., Hall, T.E.: Near field imaging at microwave and millimeter wave frequencies. In: IEEE/MTT-S Int. Microw. Symp., pp. 1693–1696 (2007). IEEE

[S7] McMakin, D.L., Sheen, D.M., Hall, T.E., Kennedy, M.O., Foote, H.P.: Biometric identification using holographic radar imaging techniques. In: Sensors, and Command, Control, Commun., and Intell. (C3I) Technol. for Homeland Secur. and Homeland Defense VI, vol. 6538, pp. 80–91 (2007). SPIE

[S8] McMakin, D.L., Sheen, D.M., Griffin, J.W., Lechelt, W.M.: Extremely high-frequency holographic radar imaging of personnel and mail. In: Sensors, and Command, Control, Commun., and Intell. (C3I) Technol. for Homeland Secur. and Homeland Defense V, vol. 6201, pp. 580–591 (2006). SPIE

[S9] Liu, H., Wang, S., Jing, H., Li, S., Zhao, G., Sun, H.: Millimeter-wave image deblurring via cycle-consistent adversarial network. Electronics **12**(3), 741 (2023)

[S10] Tao, X., Gao, H., Shen, X., Wang, J., Jia, J.: Scale-recurrent network for deep image deblurring. In: IEEE/CVF Int. Conf. Pattern Recognit., pp. 8174–8182 (2018)

[S11] Li, S., Wang, S., Amin, M.G., Zhao, G.: Efficient near-field imaging using cylindrical MIMO arrays. IEEE Trans. Aerosp. Electron. Syst. **57**(6), 3648–3660 (2021)

[S12] Ahmed, S.S., Schiessl, A., Schmidt, L.-P.: A novel fully electronic active real-time imager based on a planar multistatic sparse array. IEEE Trans. Microw. Theory Techn. **59**(12), 3567–3576 (2011)

[S13] Desai, M.D., Jenkins, W.K.: Convolution backprojection image reconstruction for spotlight mode synthetic aperture radar. IEEE Trans. Image Process. **1**(4), 505–517 (1992)

[S14] Zhuge, X., Yarovoy, A.G.: Three-dimensional near-field MIMO array imaging using range migration techniques. IEEE Trans. Image Process. **21**(6), 3026–3033 (2012)

[S15] Fromenteze, T., Yurduseven, O., Berland, F., Decroze, C., Smith, D.R., Yarovoy, A.G.: A transverse spectrum deconvolution technique for

MIMO short-range fourier imaging. IEEE Trans. Geosci. Remote Sens. **57**(9), 6311–6324 (2019)

[S16] Álvarez, Y., Rodriguez-Vaqueiro, Y., Gonzalez-Valdes, B., Mantzavinos, S., Rappaport, C.M., Las-Heras, F., Martínez-Lorenzo, J.Á.: Fourier-based imaging for multistatic radar systems. IEEE Trans. Microw. Theory Techn. **62**(8), 1798–1810 (2014)

[S17] Abbasi, M., Shayei, A., Shabany, M., Kavehvash, Z.: Fast fourier-based implementation of synthetic aperture radar algorithm for multistatic imaging system. IEEE Trans. Instrum. Meas. **68**(9), 3339–3349 (2018)

[S18] Moulder, W.F., Krieger, J.D., Majewski, J.J., Coldwell, C.M., Nguyen, H.T., Maurais-Galejs, D.T., Anderson, T.L., Dufilie, P., Herd, J.S.: Development of a high-throughput microwave imaging system for concealed weapons detection. In: IEEE Int. Symp. Phased Array Syst. Technol., pp. 1–6 (2016). IEEE

[S19] Li, S., Wang, S., An, Q., Zhao, G., Sun, H.: Cylindrical MIMO array-based near-field microwave imaging. IEEE Trans. Antennas Propag. **69**(1), 612–617 (2020)

[S20] Zhuge, X., Yarovoy, A.G.: A sparse aperture MIMO-SAR-basedUWB imaging system for concealed weapon detection. IEEE Trans. Geosci. Remote Sens. **49**(1), 509–518 (2010)

[S21] Gao, J., Qin, Y., Deng, B., Wang, H., Li, X.: Novel efficient 3D short-range imaging algorithms for a scanning 1D-MIMO array. IEEE Trans. Image Process. **27**(7), 3631–3643 (2018)

[S22] Zhu, R., Zhou, J., Jiang, G., Cheng, B., Fu, Q.: Grating lobe suppression in near range mimo array imaging using zero migration. IEEE Trans. Microw. Theory Techn. **68**(1), 387–397 (2019)

[S23] Gumbmann, F., Schmidt, L.-P.: Millimeter-wave imaging with optimized sparse periodic array for short-range applications. IEEE Trans. Geosci. Remote Sens. **49**(10), 3629–3638 (2011)

[S24] Gao, J., Deng, B., Qin, Y., Wang, H., Li, X.: An efficient algorithm for MIMO cylindrical millimeter-wave holographic 3-D imaging. IEEE Trans. Microw. Theory Techn. **66**(11), 5065–5074 (2018)

[S25] Gao, H., Li, C., Wu, S., Geng, H., Zheng, S., Qu, X., Fang, G.: Study of the extended phase shift migration for three-dimensional MIMO-SAR imaging in terahertz band. IEEE Access **8**, 24773–24783 (2020)

[S26] Wu, S., Wang, H., Li, C., Liu, X., Fang, G.: A modified Omega-K algorithm for near-field single-frequency MIMO-arc-array-based azimuth imaging. IEEE Trans. Antennas Propag. **69**(8), 4909–4922 (2021)

[S27] Li, S., Wang, S., Wu, S., Hoorfar, A., An, Q., Xing, G., Zhao, M., Zhao, G.: Millimeter-wave imaging via circular-arc mimo arrays. IEEE Trans. Microw. Theory Techn. **71**(7), 3156–3172 (2023)

[S28] Yang, B., Zhuge, X., Yarovoy, A., Ligthart, L.: UWB MIMO antenna array topology design using PSO for through dress near-field imaging. In: Eur. Microw. Conf., pp. 1620–1623 (2008). IEEE

[S29] Yang, B., Yarovoy, A., Aubry, P., Zhuge, X.: Experimental verification

of 2D UWB mimo antenna array for near-field imaging radar. In: Eur. Microw. Conf., pp. 97–100 (2009). IEEE

[S30] Zhuge, X., Yarovoy, A.: Near-field ultra-wideband imaging with two-dimensional sparse MIMO array. In: Eur. Conf. Antennas Propag., pp. 1–4 (2010). IEEE

[S31] Zhuge, X., Yarovoy, A.G.: Study on two-dimensional sparse MIMO UWB arrays for high resolution near-field imaging. IEEE Trans. Antennas Propag. **60**(9), 4173–4182 (2012)

[S32] Gonzalez-Valdes, B., Allan, G., Rodriguez-Vaqueiro, Y., Alvarez, Y., Mantzavinos, S., Nickerson, M., Berkowitz, B., Martı, J., Las-Heras, F., Rappaport, C.M., *et al.*: Sparse array optimization using simulated annealing and compressed sensing for near-field millimeter wave imaging. IEEE Trans. Antennas Propag. **62**(4), 1716–1722 (2013)

[S33] Tan, K., Wu, S., Wang, Y., Ye, S., Chen, J., Fang, G.: A novel two-dimensional sparse MIMO array topology for UWB short-range imaging. IEEE Antennas Wireless Propag. Lett. **15**, 702–705 (2015)

[S34] Tan, K., Wu, S., Wang, Y., Ye, S., Chen, J., Liu, X., Fang, G., Yan, S.: On sparse MIMO planar array topology optimization for UWB near-field high-resolution imaging. IEEE Trans. Antennas Propag. **65**(2), 989–994 (2016)

[S35] Yanik, M.E., Torlak, M.: Near-field MIMO-SAR millimeter-wave imaging with sparsely sampled aperture data. IEEE Access **7**, 31801–31819 (2019)

[S36] Cheng, Q., Liu, Y., Zhang, H., Hao, Y.: A generic spiral MIMO array design method for short-range UWB imaging. IEEE Antennas Wireless Propag. Lett. **19**(5), 851–855 (2020)

[S37] An, Q., Hoorfar, A., Lv, H., Wang, J.: Task-specific sparse MIMO array design for twri using multi-objective CMA-ES. In: General Assem. Scientific Symp. Int. Union Radio Sci., pp. 1–4 (2021). IEEE

[S38] Wang, S., Li, S., Hoorfar, A., Miao, K., Zhao, G., Sun, H.: Compressive sensing based sparse MIMO array synthesis for wideband near-field millimeter-wave imaging. IEEE Trans. Aerosp. Electron. Syst. (2023)

[S39] Wang, S., Li, S., Ren, B., Miao, K., Zhao, G., Sun, H.: Convex optimization-based design of sparse arrays for 3-D near-field imaging. IEEE Sensors J. (2023)

[S40] Ahmed, S.S., Schiess, A., Schmidt, L.-P.: Near field mm-wave imaging with multistatic sparse 2D-arrays. In: Eur. Radar Conf., pp. 180–183 (2009). IEEE

[S41] Huang, C., Wang, S., Li, S.: Modified markov random fields-based variational bayesian imaging approach for cluster structured faint scattered targets. In: Int. Conf. Microw. Millimeter Wave Technol., pp. 1–3 (2022). IEEE

[S42] Lv, M., Chen, H., Ma, J., Chen, L., Yang, J., Ma, X.: 2D high-resolution ISAR imaging by joint using matrix completion and compressed sensing. In: CIE Int. Conf. Radar, pp. 107–110 (2021)

[S43] Liu, J., Xu, S., Gao, X., Li, X.: Compressive radar imaging methods based on fast smoothed l0 algorithm. Procedia Eng. **29**, 2209–2213 (2012)

[S44] Coker, J.D., Tewfik, A.H.: Compressed sensing and multistatic SAR. In: IEEE Int. Conf. Acoust. Speech Signal Process., pp. 1097–1100 (2009). IEEE

[S45] Li, S., Zhao, G., Zhang, W., Qiu, Q., Sun, H.: ISAR imaging by two-dimensional convex optimization-based compressive sensing. IEEE Sensors J. **16**(19), 7088–7093 (2016)

[S46] Li, S., Zhao, G., Li, H., Ren, B., Hu, W., Liu, Y., Yu, W., Sun, H.: Near-field radar imaging via compressive sensing. IEEE Trans. Antennas Propag. **63**(2), 828–833 (2014)

[S47] Barzegar, A.S., Cheldavi, A., Sedighy, S.H., Nayyeri, V.: 3-D through-the-wall radar imaging using compressed sensing. IEEE Geosci. Remote Sens. Lett. **19**, 1–5 (2021)

[S48] Wang, X., Li, G., Liu, Y., Amin, M.G.: Two-level block matching pursuit for polarimetric through-wall radar imaging. IEEE Trans. Geosci. Remote Sens. **56**(3), 1533–1545 (2017)

[S49] Fang, Y., Wang, B., Sun, C., Wang, S., Hu, J., Song, Z.: Joint sparsity constraint interferometric ISAR imaging for 3-D geometry of near-field targets with sub-apertures. Sensors **18**(11), 3750 (2018)

[S50] Li, S., Zhao, G., Sun, H., Amin, M.: Compressive sensing imaging of 3-d object by a holographic algorithm. IEEE Trans. Antennas Propag. **66**(12), 7295–7304 (2018)

[S51] Ichikawa, K., Hirose, A.: Singular unit restoration in InSAR using complex-valued neural networks in the spectral domain. IEEE Trans. Geosci. Remote Sens. **55**(3), 1717–1723 (2016)

[S52] Gao, J., Deng, B., Qin, Y., Wang, H., Li, X.: Enhanced radar imaging using a complex-valued convolutional neural network. IEEE Geosci. Remote Sens. Lett. **16**(1), 35–39 (2018)

[S53] Hu, C., Wang, L., Li, Z., Zhu, D.: Inverse synthetic aperture radar imaging using a fully convolutional neural network. IEEE Geosci. Remote Sens. Lett. **17**(7), 1203–1207 (2019)

[S54] Li, R., Zhang, S., Zhang, C., Liu, Y., Li, X.: Deep learning approach for sparse aperture isar imaging and autofocusing based on complex-valued admm-net. IEEE Sensors J. **21**(3), 3437–3451 (2020)

[S55] Cheng, Q., Ihalage, A.A., Liu, Y., Hao, Y.: Compressive sensing radar imaging with convolutional neural networks. IEEE Access **8**, 212917–212926 (2020)

[S56] Su, W.-t., Hung, Y.-C., Chao, T.-H., Yu, P.-J., Yang, S.-H., Lin, C.-W.: Seeing through a black box: Toward high-quality terahertz tomographicimaging via multi-scale spatio-spectral image fusion. arXiv preprint arXiv:2103.16932 (2021)

[S57] Wang, H., Li, K., Lu, X., Zhang, Q., Luo, Y., Kang, L.: Isar resolution enhancement method exploiting generative adversarial network. Remote

Sens. **14**(5), 1291 (2022)

[S58] Yuan, H., Li, H., Zhang, Y., Wang, Y., Liu, Z., Wei, C., Yao, C.: High-resolution refocusing for defocused ISAR images by complex-valued Pix2pixHD network. IEEE Geosci. Remote Sens. Lett. **19**, 1–5 (2022)

[S59] Yuan, Y., Luo, Y., Ni, J., Zhang, Q.: Inverse synthetic aperture radar imaging using an attention generative adversarial network. Remote Sens. **14**(15), 3509 (2022)

[S60] Li, W., Yuan, Y., Zhang, Y., Luo, Y.: Unblurring ISAR imaging for maneuvering target based on UFGAN. Remote Sens. **14**(20), 5270 (2022)

[S61] Li, M., Wu, J., Huo, W., Jiang, R., Li, Z., Yang, J., Li, H.: Target-oriented SAR imaging for SCR improvement via deep MF-ADMM-Net. IEEE Trans. Geosci. Remote Sens. **60**, 1–14 (2022)

[S62] Su, Z., Zhang, Y., Zhou, J., Shi, J., Qi, F.: Data-driven based terahertz image restoration. IEEE Sensors J. (2023)

[S63] Li, L., Wang, L.G., Teixeira, F.L., Liu, C., Nehorai, A., Cui, T.J.: DeepNIS: Deep neural network for nonlinear electromagnetic inverse scattering. IEEE Trans. Antennas Propag. **67**(3), 1819–1825 (2018)

[S64] Wei, Z., Chen, X.: Deep-learning schemes for full-wave nonlinear inverse scattering problems. IEEE Trans. Geosci. Remote Sens. **57**(4), 1849–1860 (2018)

[S65] Chen, X., Wei, Z., Maokun, L., Rocca, P., *et al.*: A review of deep learning approaches for inverse scattering problems (invited review). Electromagn. Waves **167**, 67–81 (2020)

[S66] Wei, S., Liang, J., Wang, M., Zeng, X., Shi, J., Zhang, X.: CIST: An improved ISAR imaging method using convolution neural network. Remote Sens. **12**(16), 2641 (2020)

[S67] Li, X., Bai, X., Zhou, F.: High-resolution ISAR imaging and autofocusing via 2D-ADMM-Net. Remote Sens. **13**(12), 2326 (2021)

[S68] Wang, M., Wei, S., Liang, J., Zeng, X., Wang, C., Shi, J., Zhang, X.: Rmist-net: Joint range migration and sparse reconstruction network for 3-D mmW imaging. IEEE Trans. Geosci. Remote Sens. **60**, 1–17 (2021)

[S69] Wei, S., Liang, J., Wang, M., Shi, J., Zhang, X., Ran, J.: Af-ampnet: A deep learning approach for sparse aperture ISAR imaging and autofocusing. IEEE Trans. Geosci. Remote Sens. **60**, 1–14 (2021)

[S70] Xiao, C., Gao, X., Zhang, C.: U-ADMMNet: A mdel-based deep learning method for sparse aperture isar imaging. In: Int. Congr. Image Signal Process. BioMed. Eng. Inform., pp. 1–7 (2021). IEEE

[S71] Wang, M., Wei, S., Liang, J., Zhou, Z., Qu, Q., Shi, J., Zhang, X.: Tpssi-net: Fast and enhanced two-path iterative network for 3D SAR sparse imaging. IEEE Trans. Image Process. **30**, 7317–7332 (2021)

[S72] Hu, X., Xu, F., Guo, Y., Feng, W., Jin, Y.-Q.: Mdli-net: Model-driven learning imaging network for high-resolution microwave imaging with large rotating angle and sparse sampling. IEEE Trans. Geosci. Remote Sens. **60**, 1–17 (2021)

[S73] Wang, M., Wei, S., Liang, J., Liu, S., Shi, J., Zhang, X.: Lightweight FISTA-inspired sparse reconstruction network for mmw 3-D holography. IEEE Trans. Geosci. Remote Sens. **60**, 1–20 (2021)

[S74] An, H., Jiang, R., Wu, J., Teh, K.C., Sun, Z., Li, Z., Yang, J.: LRSR-ADMM-Net: A joint low-rank and sparse recovery network for SAR imaging. IEEE Trans. Geosci. Remote Sens. **60**, 1–14 (2022)

[S75] Rostami, P., Zamani, H., Fakharzadeh, M., Amini, A., Marvasti, F.: A deep learning approach for reconstruction in millimeter-wave imaging systems. IEEE Trans. Antennas Propag. **71**(1), 1180–1184 (2022)

[S76] Jiang, Y., Li, G., Ge, H., Wang, F., Li, L., Chen, X., Lv, M., Zhang, Y.: Adaptive compressed sensing algorithm for terahertz spectral image reconstruction based on residual learning. Spectrochimica Acta Part A: Mol. Biomolecular Spectrosc. **281**, 121586 (2022)

[S77] Zhou, Z., Wei, S., Zhang, H., Shen, R., Wang, M., Shi, J., Zhang, X.: SAF-3DNet: Unsupervised AMP-inspired network for 3-D MMW SAR imaging and autofocusing. IEEE Trans. Geosci. Remote Sens. **60**, 1–15 (2022)

[S78] Wei, S., Zhou, Z., Wang, M., Zhang, H., Shi, J., Zhang, X., Fan, L.: Learning-based split unfolding framework for 3-D mmW radar sparse imaging. IEEE Trans. Geosci. Remote Sens. **60**, 1–17 (2022)

[S79] Wang, M., Wei, S., Zhou, Z., Shi, J., Zhang, X.: Efficient ADMM framework based on functional measurement model for mmW 3-D SAR imaging. IEEE Trans. Geosci. Remote Sens. **60**, 1–17 (2022)

[S80] Lv, M., Chen, W., Yang, J., Wang, D., Wu, X., Ma, X.: Joint 2D sparse ISAR imaging and autofocusing by using 2D-IADIANet. IEEE Sensors J. (2023)

[S81] Bao, J., Li, D., Li, S., Zhao, G., Sun, H., Zhang, Y.: Fine-grained image generation network with radar range profiles using cross-modal visual supervision. IEEE Trans. Microw. Theory Techn. (2023)

[S82] Gubernatis, J., Domany, E., Krumhansl, J., Huberman, M.: The born approximation in the theory of the scattering of elastic waves by flaws. J. Appl. Phys. **48**(7), 2812–2819 (1977)

[S83] Loshchilov, I., Hutter, F.: Decoupled weight decay regularization. arXiv preprint arXiv:1711.05101 (2017)

[S84] Ilya, L., Frank, H.: SGDR: Stochastic gradient descent with warm restarts. In: Int. Conf. Learn. Representations (2017)

[S85] Zhou, J., Zhu, R., Jiang, G., Zhao, L., Cheng, B.: A precise wavenumber domain algorithm for near range microwave imaging by cross MIMO array. IEEE Trans. Microw. Theory Techn. **67**(4), 1316–1326 (2019)

[S86] Jocher, G., Chaurasia, A., Qiu, J.: YOLO by Ultralytics. https://github.com/ultralytics (2023)

[S87] Redmon, J., Farhadi, A.: YOLOv3: An incremental improvement. arXiv preprint arXiv:1804.02767 (2018)

[S88] Park, H., Yoo, Y., Seo, G., Han, D., Yun, S., Kwak, N.: C3: Concentrated-comprehensive convolution and its application to semantic

segmentation. arXiv preprint arXiv:1812.04920 (2018)

[S89] Ge, Z., Liu, S., Wang, F., Li, Z., Sun, J.: YOLOX: Exceeding yolo series in 2021. arXiv preprint arXiv:2107.08430 (2021)

[S90] Mao, X., Li, Q., Xie, H., Lau, R.Y., Wang, Z., Paul Smolley, S.: Least squares generative adversarial networks. In: IEEE Int. Conf. Comput. Vision, pp. 2794–2802 (2017)

[S91] Li, X., Wang, W., Wu, L., Chen, S., Hu, X., Li, J., Tang, J., Yang, J.: Generalized focal loss: Learning qualified and distributed bounding boxes for dense object detection. In: Adv. Neural Inf. Process. Syst., vol. 33, pp. 21002–21012 (2020)

[S92] Zheng, Z., Wang, P., Ren, D., Liu, W., Ye, R., Hu, Q., Zuo, W.: Enhancing geometric factors in model learning and inference for object detection and instance segmentation. IEEE Trans. Cybern. **52**(8), 8574–8586 (2021)

[S93] Bochkovskiy, A., Wang, C.-Y., Liao, H.-Y.M.: YOLOv4: Optimal speed and accuracy of object detection. arXiv preprint arXiv:2004.10934 (2020)

[S94] Zheng, L., Shen, L., Tian, L., Wang, S., Wang, J., Tian, Q.: Scalable person re-identification: A benchmark. In: IEEE Int. Conf. Comput. Vision, pp. 1116–1124 (2015)

[S95] Altair Engineering Inc.: FEKO. https://www.altairhyperworks.com/feko (2018)
